# Nerve-independent formation of membrane infoldings at topologically complex postsynaptic apparatus by caveolin-3

**DOI:** 10.1126/sciadv.adg0183

**Published:** 2023-06-16

**Authors:** Hui-Lam Rachel Kwan, Zora Chui-Kuen Chan, Xinyi Bi, Justyna Kutkowska, Tomasz J. Prószyński, Chi Bun Chan, Chi Wai Lee

**Affiliations:** ^1^ School of Biomedical Sciences, Li Ka Shing Faculty of Medicine, The University of Hong Kong, Hong Kong, China.; ^2^ School of Biological Sciences, Faculty of Science, The University of Hong Kong, Hong Kong, China.; ^3^ Łukasiewicz Research Network – PORT Polish Center for Technology Development, Wrocław, Poland.; ^4^ Golden Meditech Centre for NeuroRegeneration Sciences, Hong Kong Baptist University, Hong Kong, China.

## Abstract

Junctional folds are unique membrane specializations developed progressively during the postnatal maturation of vertebrate neuromuscular junctions (NMJs), but how they are formed remains elusive. Previous studies suggested that topologically complex acetylcholine receptor (AChR) clusters in muscle cultures undergo a series of transformations, resembling the postnatal maturation of NMJs in vivo. We first demonstrated the presence of membrane infoldings at AChR clusters in cultured muscles. Live-cell super-resolution imaging further revealed that AChRs are gradually redistributed to the crest regions and spatially segregated from acetylcholinesterase along the elongating membrane infoldings over time. Mechanistically, lipid raft disruption or caveolin-3 knockdown not only inhibits membrane infolding formation at aneural AChR clusters and delays agrin-induced AChR clustering in vitro but also affects junctional fold development at NMJs in vivo. Collectively, this study demonstrated the progressive development of membrane infoldings via nerve-independent, caveolin-3–dependent mechanisms and identified their roles in AChR trafficking and redistribution during the structural maturation of NMJs.

## Teaser

Nerve-independent membrane infoldings in muscle cultures mirror junctional folds at postsynaptic apparatus at NMJs in vivo.

## INTRODUCTION

The vertebrate neuromuscular junction (NMJ), a peripheral chemical synapse between a motor neuron and a skeletal muscle fiber, is essential for all our voluntary movement. At NMJs, acetylcholine receptor (AChR) clusters are highly enriched at the postsynaptic membrane, making chemical neurotransmission efficient (*1*, *2*). Early ultrastructural studies revealed that large protein complexes of active zone components perfectly align with the junctional folds at adult NMJs (*3*, *4*), suggesting the instructive role of motor nerve innervation in postsynaptic junctional fold development. Such topological membrane specializations in the postsynaptic apparatus are gradually developed by a series of transformations initiated from the formation of a gutter, and subsequently, the gutter membrane invaginates to form a number of secondary folds during the early postnatal stages (*5*). Apart from using electron microscopy, the spatial enrichment of AChRs at the crests of junctional folds can also be detected at mature NMJs using superresolution imaging approaches (*6*). In contrast, the trough regions of the folds contain a high concentration of voltage-gated sodium channels (VGSCs), which are spatially segregated from the adjacent AChR clusters (*7*). However, the molecular mechanisms underlying the formation of membrane folds with spatially segregated postsynaptic proteins in NMJ development remain largely unclear.

Even in the absence of motor neurons, aneural AChR clusters can still be found at the center regions of muscle fibers in vivo (*8*, *9*). These spontaneously formed AChR clusters are believed to be regulated by muscle-intrinsic mechanisms. Similar topologically complex aneural AChR cluster structures can be induced in the basal membrane of different primary muscle cultures (*Xenopus*, mouse, and human) and immortalized C2C12 myotubes when they are cultured on extracellular matrix (ECM) protein laminin-containing substrates (*10*–*12*). These ECM-induced aneural AChR clusters in muscle cultures can undergo a similar developmentally regulated topological transformation from a simple oval plaque-shaped to elaborated perforated, pretzel-shaped, and C-shaped clusters as seen in vivo (*10*). Podosome-like structures (PLSs), which are highly enriched at the AChR-poor perforations of ECM-induced aneural AChR clusters in cultured muscles, are implicated in, and likely accelerate, the topological transformation and maturation of postsynaptic apparatus normally occurring at early postnatal development in mice (*10*, *13*). However, whether membrane folds, one of the hallmarks of NMJ maturation, are also developed in these aneural AChR clusters remains unknown.

At developing NMJs, lipid rafts are known to serve as a signaling platform to regulate the intracellular trafficking of rapsyn and AChRs to the postsynaptic membrane (*14*, *15*). Caveolae, a subset of lipid rafts, are unique invaginated membrane nanodomains that play a major role in organizing cellular signaling pathways, lipid homeostasis, and adaptation to membrane tension (*16*). The muscle-specific isoform, caveolin-3, is colocalized with AChR clusters and is required for AChR clustering in cultured myotubes through muscle-specific kinase (MuSK)–Rac1 signaling (*17*, *18*). However, whether lipid rafts and caveolin-3 regulate the formation of membrane infoldings, which is distinct from their known functions in AChR clustering, remains to be investigated.

In this study, we performed Airyscan confocal superresolution imaging studies to demonstrate the presence of membrane infolding structures associated with topologically complex aneural AChR clusters in cultured muscles without motor nerve innervation. These stably maintained membrane infoldings elongated over time and subsequently exhibited spatial enrichment of AChRs at the crest regions of the folds, resembling the structural features of the postsynaptic apparatus observed at mature NMJs in vivo. We found that lipid raft integrity and precise endogenous caveolin-3 expression levels are essential for the formation of membrane infoldings at ECM-induced aneural AChR clusters in muscle cultures and the development of junctional folds at postnatal mouse NMJs in vivo, different from their well-characterized roles in AChR clustering. In *Xenopus* nerve-muscle co-cultures, caveolin-3 knockdown showed a dose-dependent inhibition of nerve- and agrin-induced synaptic AChR clustering by attenuating AChR endocytosis and recruitment from aneural clusters with reduced membrane infoldings. Together, this study revealed a nerve-independent, caveolin-3–dependent mechanism underlying membrane infolding formation at aneural and synaptic AChR clusters and a functional role of membrane infoldings in serving as a hub for vesicular trafficking to facilitate AChR redistribution and recruitment during the structural maturation of postsynaptic apparatus.

## RESULTS

### Membrane infoldings are associated with ECM-induced aneural AChR clusters

**Fig. 1.**
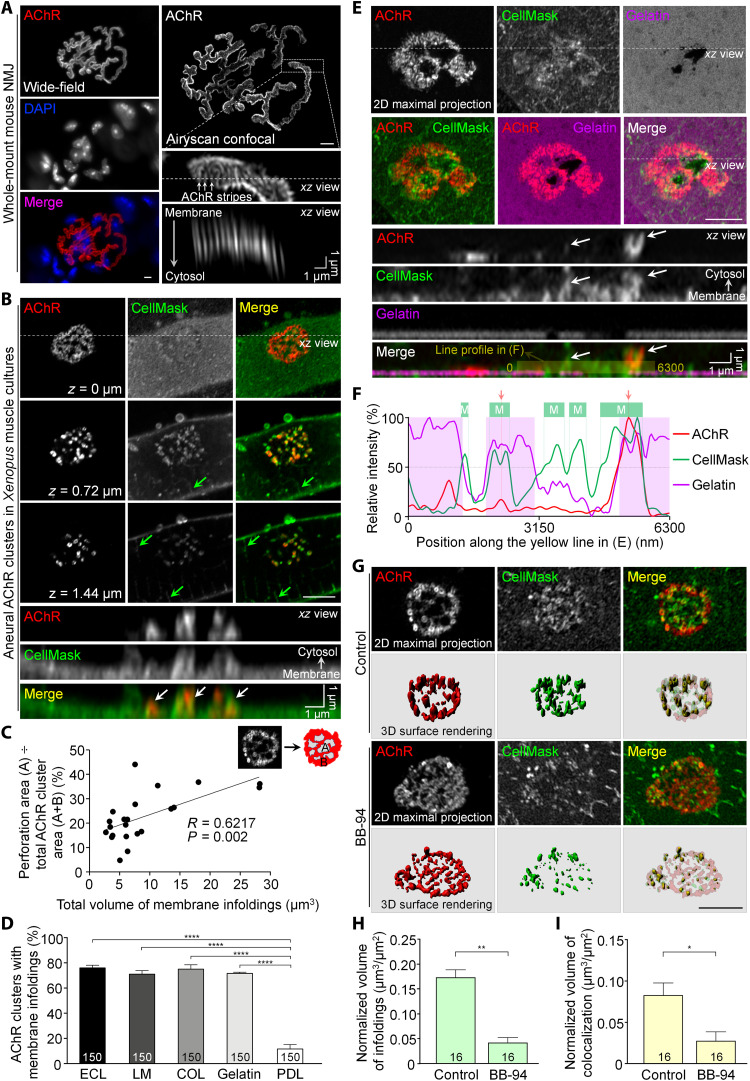
ECM proteins and their remodeling are required for membrane infolding formation at topologically complex AChR clusters. (**A**) Airyscan confocal images showing the ultrastructural organization of the postsynaptic apparatus at mature mouse NMJs. Junctional folds are identified as AChR stripes (arrows) in the maximal and *xz* orthogonal projection images. (**B**) Different focal planes of Airyscan confocal images showing the association of membrane infoldings with aneural AChR clusters in cultured *Xenopus* muscle cells. White arrows, spatial colocalization of AChR and membrane infoldings; green arrows, T-tubules. (**C**) A scatter plot showing the correlation between the perforated area of AChR clusters and the total membrane infolding volume. (**D**) Quantification showing the percentage of aneural AChR clusters with membrane infoldings in muscle cells cultured on different substrates. LM, laminin; COL, collagen. (**E**) Representative confocal *z*-stack images showing the spatial association of membrane infolding and ECM degradation at aneural AChR clusters. Arrows, AChR localization at membrane infoldings; yellow line, line profile generation in (F). (**F**) A line profile showing the relative intensities of AChR, CellMask, and fluorescent gelatin along the yellow line in (E). Shaded regions were highlighted by a 50% cutoff intensity. Red arrows mark AChR peaks. (**G**) Maximal projection and surface rendering confocal images showing the effects of BB-94 on membrane infolding formation in aneural AChR clusters. (**H** and **I**) Quantification showing the effects of BB-94 on the normalized volume of membrane infoldings (H) and the colocalization volume of AChRs and membrane infoldings (I) per unit area of aneural clusters. Scale bars, 5 μm (unless stated otherwise). Data are means ± SEM. The numbers indicated in the bar regions represent the total numbers of muscle cells quantified from three independent experiments. *P ≤ 0.05, **P ≤ 0.01, and ****P ≤ 0.0001 [one-way analysis of variance (ANOVA) with Dunnett’s multiple comparisons test (D) and Student’s *t* test (H) and (I)].

The ultrastructural organization of mature NMJs has recently been revealed by structured illumination microscopy, which convincingly indicates AChR enrichment at the crest regions of junctional folds as AChR stripes within the topologically complex postsynaptic apparatus (*6*). In this study, we used Airyscan confocal super-resolution microscopy that enables high-speed imaging in live cells (*19*), by which we also detected similar AChR stripes in the maximal projection images of whole-mount NMJs from adult mice (Fig. 1A). In addition, the spatial distribution of AChRs along the junctional folds could also be clearly observed in the orthogonal projection of *z*-stack images. Growing evidence suggests that ECM-induced aneural AChR clusters exhibit a similar topological transformation from a simple plaque to more elaborated (e.g., perforated, pretzel-shaped, and C-shaped) structures as seen during the maturation of the postsynaptic apparatus at NMJs in vivo (*10*). Here, we first used a vital membrane dye CellMask, which allows fast and uniform labeling of the plasma membrane in live cells, to examine whether membrane specializations are present at ECM-induced aneural AChR clusters in cultured *Xenopus* muscles. Using Airyscan confocal super-resolution microscopy, CellMask signals were detected in the entire plasma membrane, as well as the developing sarcolemma invaginations at the transverse-tubules (T-tubules) (Fig. 1B, green arrows). We also frequently observed membrane infolding structures (approximately 1 to 2 μm in length, white arrows in the orthogonal *xz* view) at the sites of topologically complex perforated aneural AChR clusters in 2- or 3-day-old *Xenopus* muscle cells cultured on substrata coated with a mixture of several ECM proteins, including entactin/nidogen, collagen, and laminin (ECL). Moreover, we found that membrane infoldings were progressively developed at perforated aneural AChR clusters in muscle cells over the first 4 days in culture (fig. S1, A and B). In contrast, simple oval plaque-shaped AChR clusters showed fewer or no membrane infoldings (fig. S1C). Because PLSs are spatially enriched at AChR-poor perforations of aneural clusters (*20*), we measured the percentage of perforation area in the whole AChR clusters to reflect the abundance of PLS localization (Fig. 1C). By analyzing 22 aneural AChR clusters in *Xenopus* muscles from three independent experiments, we detected a positive correlation between the percentage of perforation area and the volume of membrane infoldings within the aneural clusters, indicating the possible correlation between PLS localization and membrane infolding formation. Our recent study showed that topologically complex structures of PLS-associated aneural AChR clusters can be induced in the basal membrane of cultured *Xenopus* muscle cells by different ubiquitously expressed ECM proteins (*20*). Therefore, we also tested the ability of these ubiquitous ECM proteins (including laminin, collagen, and gelatin) to induce membrane infolding formation in cultured muscle cells. We detected extensive membrane infoldings at perforated AChR clusters in muscle cells cultured on all individual ECM proteins tested. In contrast, the negative control poly-d-lysine (PDL), which only promotes cell attachment, was found to be ineffective in inducing the formation of topologically complex aneural AChR clusters and their associated membrane infoldings (Fig. 1D). Similar to *Xenopus* primary muscle cultures, more extensive membrane infoldings were observed in perforated and C-shaped AChR clusters, compared with plaque-shaped AChR clusters, in C2C12 myotubes cultured on laminin-coated substratum (fig. S2).

We previously demonstrated that matrix metalloproteinase (MMP)–mediated ECM degradation is detected at PLS-enriched perforations of aneural AChR clusters (*20*). Here, we also observed that membrane infoldings were spatially associated with the perforation sites of aneural AChR clusters with extensive focal degradation of fluorescent gelatin (Fig. 1, E and F), suggesting that dynamic ECM remodeling may modulate substrate stiffness to promote membrane infolding formation. Next, we incubated muscle cells with the broad-spectrum MMP inhibitor BB-94 at 5 μM, which completely abolished PLS-associated focal ECM degradation at perforated AChR clusters in our previous study (*20*). To clearly depict the presence of membrane infoldings within aneural AChR clusters, we performed surface rendering and signal filtering processing on three-dimensional (3D) images (fig. S3), by which we showed a significant reduction in membrane infoldings associated with aneural AChR clusters in response to BB-94 treatment (Fig. 1G). Quantitative analyses further indicated that BB-94 treatment significantly reduced the volume of membrane infoldings (Fig. 1H) and the colocalization volume between AChR clusters and membrane infoldings (Fig. 1I). Together, these data demonstrated the requirement of MMP-mediated ECM remodeling for the formation and/or maintenance of membrane infoldings at aneural AChR clusters.

### AChRs are gradually redistributed to the crest of membrane infoldings in live muscle cells

**Fig. 2.**
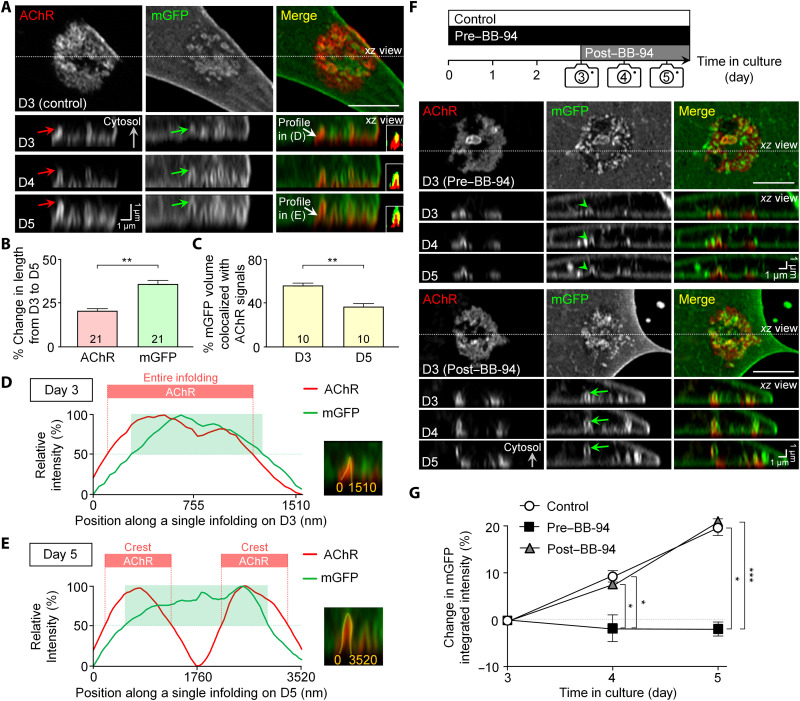
ECM remodeling is required for the progressive elongation of the same membrane infoldings monitored over time. (**A**) Representative Airyscan confocal images showing the progressive elongation of membrane infoldings at the same aneural AChR clusters between days 3 and 5 in cultures. Red and green arrows, the progressive elongation of a membrane infolding; white arrows, the membrane infolding for generating line profiles in (D) and (E). Insets: Pseudocolor merge images indicating lower AChR abundance at the tip (trough) of membrane infoldings at later time points. (**B** and **C**) Quantification showing the progressive increase in the lengths of AChR and mGFP-labeled membrane infoldings (B) and the reduced degree of their colocalization (C) between days 3 and 5 in cultures. (**D** and **E**) Line profile plots showing the relative intensities of AChR (red) signals along mGFP-labeled membrane infoldings (green) indicated in (A) on day 3 (D) and day 5 (E) muscle cultures. Shaded regions were highlighted by a 50% cutoff intensity. Yellow lines, the same single-membrane infolding identified on days 3 and 5 for generating line profiles. (**F** and **G**) Experimental timeline, representative confocal images (F), and quantification (G) showing the differential effects of BB-94 pre- and post-treatment on membrane infolding maintenance and elongation in the same aneural AChR clusters over 5 days in culture. Arrowheads, the inhibition of membrane infolding elongation by BB-94 pre-treatment. Arrows, the progressive elongation of a membrane infolding in BB-94 post-treatment group. Scale bars, 5 μm (unless stated otherwise). The numbers indicated in the bar regions represent the total numbers of membrane infoldings (B) or muscle cells (C) quantified from three independent experiments. *n* = 17 (control); *n* = 17 (pre–BB-94); *n* = 21 (post–BB-94) muscle cells from three independent experiments (G). *P ≤ 0.05, **P ≤ 0.01, and ***P ≤ 0.001 [Student’s *t* test (B) and (C) and two-way ANOVA with Dunnett’s multiple comparisons test (G)].

To study the temporal changes of membrane infoldings in correlation with AChR distribution in live muscle cells, we overexpressed a membrane-bound form of green fluorescent protein (mGFP) containing a palmitoylation sequence of growth-associated protein 43 (GAP43) at its N terminus (*21*), which allows us to study the dynamic changes of membrane infoldings in the same AChR clusters at multiple time points over a few days. In Airyscan confocal super-resolution images, mGFP-labeled membrane infoldings were frequently observed in close proximity to the perforations of aneural AChR clusters in 3-day-old live muscle cells (Fig. 2A), in agreement with the CellMask staining data above. By measuring the progressive development of mGFP-labeled membrane infoldings individually, we detected a larger increase in the length of membrane infoldings (~36%) than that of AChR (~21%) between days 3 and 5 (Fig. 2B). At the same time, the degree of colocalization between AChRs and mGFP-labeled membrane infoldings was significantly reduced (Fig. 2C). These suggested that AChRs are initially distributed along the entire membrane infolding structures, but they are found to be less abundant at the tip (i.e., trough regions) of membrane infoldings over the next 2 days. This temporal change in AChR distribution at membrane infoldings was better appreciated by plotting line profiles of AChR and mGFP signals along a single-membrane infolding structure (Fig. 2, D and E). By using a cutoff intensity of 50%, AChRs (red-shaded regions) were found to be distributed initially along the entire membrane infolding structures (green-shaded regions) and subsequently localized preferentially at the base (i.e., crest regions) of membrane infoldings over time, resembling the spatial enrichment of AChR clusters at the crests of junctional folds at mature NMJs in vivo.

To determine the differential requirement of MMP activity in the formation and/or maintenance of membrane infoldings at aneural AChR clusters in live muscle cells, we added BB-94 either before cell plating on day 0 (pre–BB-94) or after the formation of AChR clusters with membrane infoldings on day 3 (post–BB-94) and then followed by the progressive changes of membrane infoldings at the same AChR clusters for two consecutive days (Fig. 2F). Consistent with the CellMask staining data above (Fig. 1, G to I), here, we also found that BB-94 pre-treatment caused a significant reduction in the volume of mGFP-labeled membrane infoldings at the same AChR clusters in live muscle cells (Fig. 2, F and G). In addition, the elongation of the existing membrane infoldings found on day 3 was also largely inhibited by BB-94 pre-treatment (green arrowheads). In contrast, we observed a gradual increase in the length of the same mGFP-labeled membrane infoldings (green arrows) in both the untreated control and post–BB-94–treated groups. These results indicated that MMP activity during the initial stage of aneural AChR cluster formation enables the subsequent elongation and maintenance of the associated membrane infoldings.

**Fig. 3.**
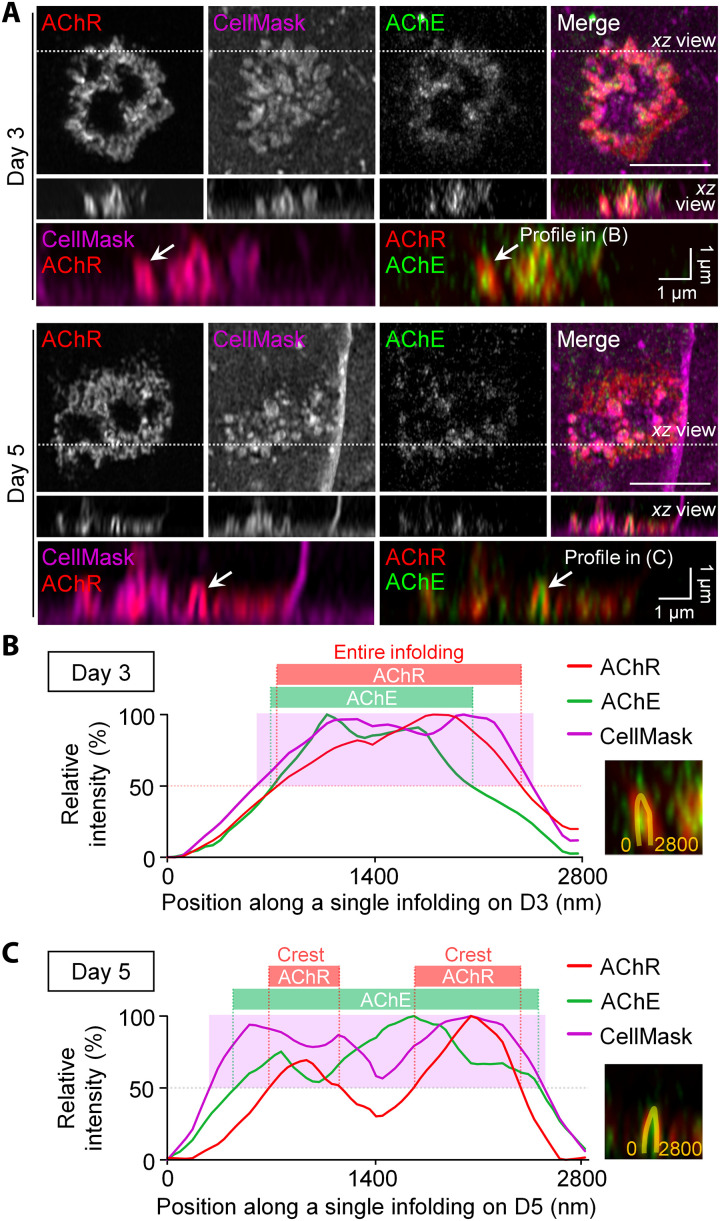
Time-dependent elongation of membrane infoldings exhibits differential localization of AChR and AChE along the length of membrane infoldings. (**A**) Maximal and orthogonal projection of Airyscan confocal *z*-stack images showing the spatial enrichment of AChE along the entire membrane infolding region at aneural AChR clusters in both 3- and 5-day-old live muscle cells. Arrows, the spatial distribution of AChR and AChE along the CellMask-labeled membrane infoldings in the orthogonal views. (**B** and **C**) Line profile plots showing the relative intensities of AChR (red), AChE (green), and CellMask (magenta) signals at aneural AChR clusters in 3- (B) and 5-day-old (C) muscle cells. The spatial distribution of AChR and AChE along the CellMask-labeled membrane infolding was highlighted in shaded regions by a 50% cutoff intensity. Yellow lines in the insets indicate the regions of interest (ROIs) for generating line profiles. Scale bars, 5 μm (unless stated otherwise).

To further test the hypothesis that membrane infoldings at aneural AChR clusters resemble the junctional folds at mature NMJs, we next examined the spatial localization of endogenous postsynaptic proteins along different domains of membrane infoldings at aneural AChR clusters. Contrary to a previous study indicating an absence of acetylcholinesterase (AChE) at aneural AChR clusters in cultured C2C12 myotubes (*10*), we detected a spatial enrichment of AChE at aneural AChR clusters in primary *Xenopus* muscle cultures (Fig. 3A). In 3-day-old muscle cells, AChR and AChE were both distributed along the entire membrane infolding in aneural AChR clusters (Fig. 3, A and B). Consistent with the data above, AChRs were gradually redistributed to and preferentially localized at the crest regions of membrane infoldings in 5-day-old muscle cells; however, AChE remained distributed along the entire membrane infolding structure (Fig. 3, A and C). Such spatial organization of AChR and AChE at aneural clusters that we observed in cultured *Xenopus* muscle cells resembles the ultrastructural organization of postsynaptic junctional folds at mammalian NMJs as revealed by electron microscopy images (*22*). Together, our data support the hypothesis that the membrane infoldings at aneural AChR clusters morphologically and molecularly resemble the junctional folds at mature NMJs in vivo.

### Lipid rafts and caveolin-3 are required for the formation of membrane infoldings

**Fig. 4.**
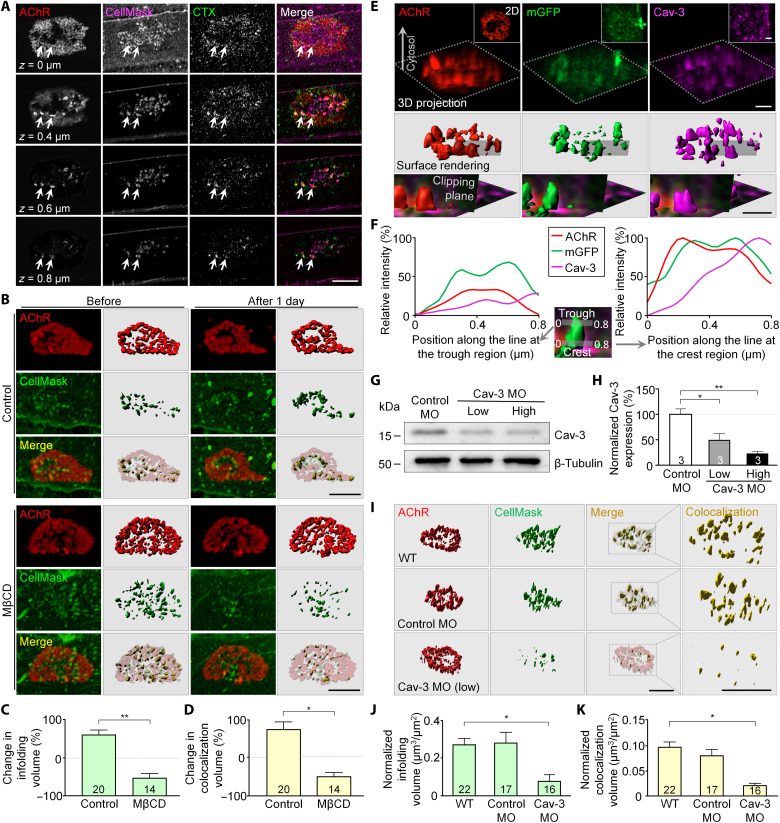
Lipid rafts and caveolin-3 regulate membrane infolding formation and maintenance at aneural AChR clusters. (**A**) Different focal planes of Airyscan confocal images showing CTX-labeled lipid rafts at aneural AChR clusters (arrows). (**B**) Representative 3D and surface rendering images showing the effects of MβCD treatment on membrane infolding formation and maintenance. For clarity, only signals at aneural AChR clusters were included in the surface rendering images. (**C** and **D**) Quantification showing the effects of MβCD treatment on the percentage changes in membrane infolding volume (C) and colocalization volume of AChRs and membrane infoldings (D) after 1 day. (**E**) Representative images showing the spatial localization of endogenous caveolin-3 at membrane infoldings. Insets: 2D maximal projection images. Bottom row: The magnified view of different markers in a clipping (dark vertical) plane to better visualize a single-membrane infolding. (**F**) Line profile plots showing the spatial enrichment of AChR and caveolin-3 at the crest (right chart), but less at the trough (left chart), regions along a single mGFP-labeled membrane infolding. (**G** and **H**) Western blot analysis (G) and quantification (H) showing the effective knockdown of endogenous caveolin-3 expression by antisense caveolin-3 MO. (**I**) Representative images showing the effects of caveolin-3 knockdown on the formation of membrane infoldings. 2D maximal projection images are included in fig. S9B. (**J** and **K**) Quantification showing the normalized volume of membrane infoldings (J) and colocalization volume of AChRs and membrane infoldings (K) per unit area of aneural clusters. Scale bars, 5 μm (A), (B), and (I) or 1 μm (E). Data are means ± SEM. The numbers indicated in the bar regions represent the total numbers of muscle cells (C), (D), (J), and (K) or blots (H) quantified from three independent experiments. *P ≤ 0.05 and **P ≤ 0.01 [Student’s *t* test (C) and (D) and one-way ANOVA with Dunnett’s multiple comparisons test (H), (J), and (K)].

Lipid rafts are concentrated at postsynaptic specializations and are colocalized with AChR clusters in cultured myotubes (*14*, *23*). To investigate whether lipid rafts are involved in the formation of membrane infoldings, independent of their well-known function in AChR clustering, we first used a low dose of fluorescent cholera toxin subunit B (CTX) at 1 μg/ml to only label a fraction of the raft-specific lipid ganglioside GM1 molecules, so as to minimize the cross-linking–induced phase separation of lipid rafts (*24*). In 3-day-old muscle cultures, fluorescent CTX signals were found to be spatially enriched at sites of membrane infoldings within aneural AChR clusters as observed in different focal planes of confocal *z*-stack images (Fig. 4A). Next, we used methyl-β-cyclodextrin (MβCD), which acutely depletes cholesterol from the plasma membrane (*25*), to investigate the effects of lipid raft disruption on the formation and/or maintenance of membrane infoldings at aneural AChR clusters. In muscle cells pre-treated with MβCD, we only detected plaque-shaped aneural AChR clusters with no membrane infoldings (fig. S4). Then, MβCD was added to 2-day-old muscle cultures to examine the requirement of lipid rafts for both the maintenance/elongation of the existing membrane infoldings and the formation of the new ones within the aneural AChR clusters. In control muscle cells, we observed an increased volume (+54.7 ± 15.7%) of membrane infoldings at the same AChR clusters after 1 day (Fig. 4, B and C). In contrast, MβCD post-treatment resulted in a significant reduction in membrane infolding volume (−57.1 ± 12%) in the same AChR clusters. In addition, 3D surface rendered images showed a significant reduction in the number and size of membrane infoldings, leading to reduced colocalization of AChR and CellMask signals, after MβCD treatment (Fig. 4, B and D). Different from the drastic inhibitory effects of lipid raft disruption on AChR clustering in previous studies (*14*, *15*), our data demonstrated that lipid raft integrity is crucial for the formation, maintenance, and elongation of membrane infoldings in live muscle cultures, even when AChR clustering is largely unaffected.

Caveolae are well-characterized subdomains of lipid rafts involved in the formation of invaginations in the plasma membrane (*26*). Caveolin-3, a muscle-specific caveolin family member, is associated with sarcolemma invaginations at the T-tubule (*27*) and is highly localized at the NMJs (*17*, *18*, *28*). Here, we performed immunostaining experiments in mGFP-expressing muscle cells to examine whether endogenous caveolin-3 is localized at membrane infoldings within aneural AChR clusters. Airyscan confocal super-resolution images with surface rendering processing and line profile analyses showed that caveolin-3 molecules were concentrated at sites of membrane infoldings (Fig. 4E), particularly at the crest regions with enriched AChR signals (Fig. 4F). By quantifying the colocalization between caveolin-3 and mGFP signals, we detected caveolin-3 signals enriched at 44.96 ± 2.64% of the total volume of membrane infoldings. To further determine the requirement of caveolin-3 in membrane infolding formation, we used a specific antisense morpholino oligonucleotide (MO) sequence that showed a dose-dependent knockdown of endogenous caveolin-3 expression in *Xenopus* muscle tissues (Fig. 4, G and H). Because a high dose (19 ng per embryo) of caveolin-3 MO completely abolished aneural AChR cluster formation, most of our subsequent experiments used a lower dose (12 ng per embryo) for investigating membrane infoldings at aneural AChR clusters (fig. S5, A and B). Caveolin-3 immunostaining also indicated a significant reduction in endogenous caveolin-3 protein expression at aneural AChR clusters (fig. S5, C and D). By examining the aneural AChR clusters that were identified in the low caveolin-3 knockdown muscle cells, we detected fewer membrane infoldings compared with those clusters in the wild-type or control MO muscle cells (Fig. 4I). Quantitative analyses further indicated that the low dose of caveolin-3 MO drastically reduced the normalized membrane infolding volume and the colocalization volume of AChR clusters and membrane infoldings (Fig. 4, J and K) but not AChR clustering. Collectively, these results demonstrated the essential roles of lipid raft integrity and precisely controlled endogenous caveolin-3 expression levels in the formation of membrane infoldings at ECM-induced aneural AChR clusters in muscle cells.

### Caveolin-3 is required for the formation of membrane infoldings and synaptic AChR clusters induced by synaptogenic stimulation

**Fig. 5.**
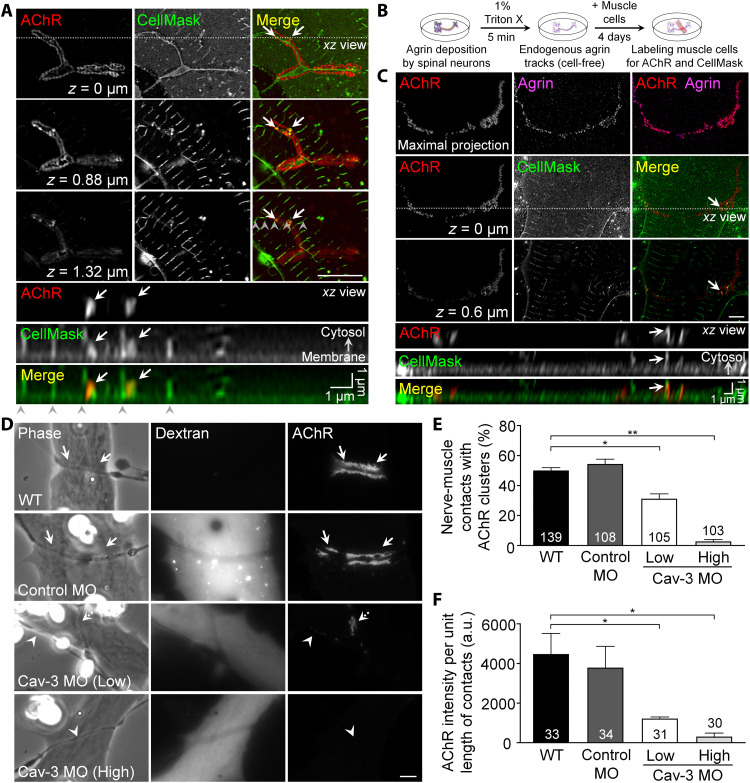
Caveolin-3 knockdown inhibits the formation of nerve-induced synaptic AChR clusters with membrane infoldings. (**A**) Different focal planes of Airyscan confocal *z*-stack images showing the presence of membrane infoldings associated with synaptic AChR clusters in nerve-muscle co-cultures. *Z* values indicate the vertical distance from the basal muscle surface. Arrows indicate the spatial colocalization of AChR clusters and membrane infoldings at nerve-muscle contacts. Arrowheads indicate the sarcolemma invaginations at the T-tubules. (**B**) Experimental procedures for the preparation of endogenous agrin tracks to induce synaptic AChR clustering in cultured muscle cells. (**C**) 2D maximal projection and different focal planes of Airyscan confocal *z*-stack images showing the presence of membrane infoldings associated with AChR clusters induced by endogenous agrin tracks. *Z* values indicate the vertical distance from the basal muscle surface. Arrows indicate the spatial colocalization of AChR clusters and membrane infoldings. (**D**) Representative images showing a dose-dependent effect of muscle caveolin-3 knockdown on nerve-induced AChR clustering. Fluorescent dextran signals indicate the presence of MO in muscle cells. Arrows indicate synaptic AChR clusters at nerve-muscle contacts. Arrowheads and a dashed arrow indicate no and reduced AChR clusters at the nerve-muscle contacts, respectively. (**E** and **F**) Quantification showing the effects of muscle caveolin-3 knockdown on the percentage of nerve-muscle contacts with AChR clusters (E) and AChR intensity per unit length of nerve-muscle contacts (F) in the chimeric co-cultures. Scale bars, 5 μm (unless stated otherwise). Data are means ± SEM. The numbers indicated in the bar regions represent the total numbers of nerve-muscle contacts measured from four (E) or three (F) independent experiments. *P ≤ 0.05 and **P ≤ 0.01 (one-way ANOVA with Dunnett’s multiple comparisons test). a.u., arbitrary units.

Next, we examined whether membrane infoldings are also present at nerve-induced synaptic AChR clusters in *Xenopus* nerve-muscle co-cultures. By using CellMask staining, we observed membrane infoldings at the contacts between the axons of spinal neurons and the basal muscle membranes on ECM-coated substratum (Fig. 5A). Orthogonal projections of Airyscan confocal *z*-stack images revealed that these membrane infoldings at nerve-muscle contacts are 1 to 2 μm in length, similar to those in aneural AChR clusters. As CellMask labels the plasma membrane of both presynaptic neurons and postsynaptic muscle cells in the co-cultures, we adopted our recently developed endogenous agrin track assay (*20*), which involves detergent extraction and removal of neurites after deposition of endogenously secreted agrin onto the substratum (Fig. 5B). After plating muscle cells on endogenous agrin tracks for 4 days, membrane infoldings were observed at synaptic AChR clusters induced by cell-free endogenous agrin tracks (Fig. 5C), suggesting that those membrane infoldings detected at nerve-muscle contacts are likely derived from postsynaptic muscle cells but not the filopodia from presynaptic neurons. To determine whether caveolin-3 is also required for the formation of nerve-induced AChR clusters with membrane infoldings, we prepared chimeric co-cultures composed of either control or caveolin-3 MO muscle cells and wild-type spinal neurons (Fig. 5D). In contrast to the extensive synaptic AChR clusters along the trails of nerve-muscle contacts in the control co-cultures, MO-mediated caveolin-3 knockdown in muscle cells significantly inhibited synaptic AChR clustering induced by wild-type spinal neurons in a dose-dependent manner (Fig. 5, E and F). In particular, a high dose of caveolin-3 MO completely abolished the formation of nerve-induced AChR clusters and their associated membrane infoldings. Together, our results demonstrated that endogenous caveolin-3 expression levels are critical for the formation of membrane infoldings at aneural AChR clusters, which, in turn, contributes to the formation of nerve-induced AChR clusters and their associated membrane infoldings.

### Caveolin-3 knockdown delays agrin-induced AChR redistribution and clustering by attenuating AChR endocytosis at aneural clusters

**Fig. 6.**
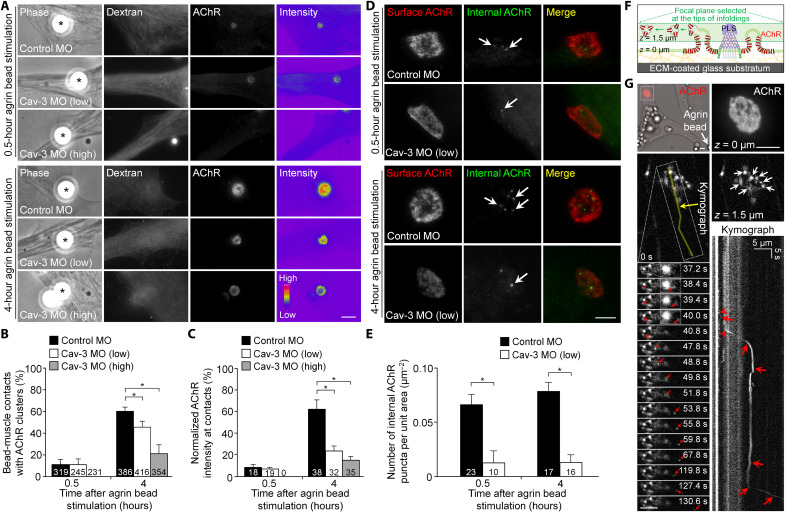
Caveolin-3 knockdown delays agrin-induced AChR clustering by attenuating AChR endocytosis at aneural clusters. (**A**) Representative images showing the effects of caveolin-3 knockdown on agrin bead–induced AChR clustering upon 0.5- or 4-hour stimulation. Fluorescent dextran signals indicate the presence of MO in muscle cells. 8-bit pseudocolor images highlight the relative fluorescence intensity of agrin bead–induced AChR clusters under different conditions. (**B** and **C**) Quantification showing the effects of caveolin-3 knockdown on the percentage of bead-muscle contacts with AChR clusters (B) and the normalized fluorescence intensity of bead-induced AChR clusters (C). (**D** and **E**) Representative images (D) and quantification (E) showing the effects of low caveolin-3 knockdown on AChR internalization at aneural clusters upon agrin bead stimulation. Arrows indicate internalized AChR vesicles at aneural clusters. (**F**) Schematic diagram illustrating the experimental approach for visualizing and tracking the formation of AChR-containing vesicles at membrane infoldings within ECM-induced aneural AChR clusters by adjusting the focal plane of high-speed time-lapse imaging at ~1.5 μm above the basal muscle membrane. (**G**) Representative time-lapse images capturing the formation of AChR-containing vesicles (red arrows) derived from the tip of membrane infoldings (red arrowheads) in agrin bead–stimulated muscle cells. A kymograph was constructed along the indicated yellow line that shows the initial formation of AChR vesicles from membrane infoldings at aneural AChR clusters, followed by directional movement toward the location of agrin bead stimulation. White arrows, the tip of membrane infoldings detected at the specified focal plane. The time-lapse video, movie S1, is available in the Supplementary Materials. Scale bars, 5 μm. Data are means ± SEM. The numbers indicated in the bar regions represent the total numbers of bead-muscle contacts (B) and (C) and bead-contacted muscle cells (E) measured from four (B) and (C) and three (E) independent experiments, respectively. *P ≤ 0.05 (one-way ANOVA with Dunnett’s multiple comparisons test).

Upon synaptogenic induction, AChRs are recruited from the aneural clusters to the synaptic sites for the assembly of postsynaptic specializations (*20*). Therefore, we hypothesized that caveolin-3–dependent membrane infolding formation may facilitate AChR redistribution from aneural to synaptic sites by vesicular trafficking regulation. To locally induce postsynaptic differentiation in muscle cells, we used latex beads coated with recombinant agrin that allowed us to induce postsynaptic differentiation in a spatially and temporally controllable manner (*11*). At 0.5 hours after agrin bead stimulation, we started to detect AChR clusters at agrin bead contacts in control MO and low dose of caveolin-3 MO muscle cells (Fig. 6A). However, no AChR clusters were detected at the bead contacts in muscle cells with a high dose of caveolin-3 MO. At 4 hours after agrin bead stimulation, both low and high doses of caveolin-3 MO caused a reduced percentage of bead-muscle contacts with AChR clusters and their intensity, compared with control MO muscle cells (Fig. 6, B and C). These data further indicated the dose-dependent inhibitory effects of caveolin-3 knockdown on membrane infolding formation at aneural AChR clusters (by low caveolin-3 MO) and aneural AChR cluster formation (by high caveolin-3 MO), which, in turn, delayed the assembly of agrin-induced synaptic AChR clusters.

It has been suggested that membrane infoldings serve as endocytic and exocytic sites for intracellular trafficking events (*29*). We next hypothesized that the delay of agrin-induced AChR clustering in caveolin-3 MO muscle cells is due to the attenuation of AChR trafficking and recruitment from aneural AChR clusters with reduced membrane infoldings. To test this, we performed a sequential labeling experiment to differentiate surface and internal AChRs using different fluorescent α-bungarotoxin (BTX) probes (*11*), by which we found that the low dose of caveolin-3 MO significantly reduced the number of AChR-containing vesicles at aneural AChR clusters upon agrin bead simulation for 0.5 and 4 hours (Fig. 6, D and E), while no AChR vesicles were detected in the high dose of caveolin-3 MO, as it completely abolished the formation of aneural AChR clusters (fig. S5, A and B). To determine whether AChR-containing vesicles are derived from the membrane infoldings at aneural AChR clusters, we performed high-speed time-lapse imaging (200 ms per frame) at a single focal plane focusing on the tip of membrane infoldings at aneural AChR clusters in agrin bead–stimulated muscle cells (Fig. 6F). In this example, time-lapse montage images and kymograph analyses revealed that two AChR-containing vesicles budded off from the tip of membrane infoldings at 38.4 and 47.8 s, followed by directional movement toward the site of agrin bead stimulation (Fig. 6G and movie S1). Together, our results suggest that precisely controlled endogenous caveolin-3 expression regulates membrane infolding formation at aneural AChR clusters, and such membrane infoldings may act as a hub for vesicular trafficking of some AChR molecules during the redistribution and recruitment of aneural clusters to nerve-induced postsynaptic specializations at developing NMJs.

### Lipid raft disruption or caveolin-3 knockdown inhibits junctional fold development at NMJs in vivo

**Fig. 7.**
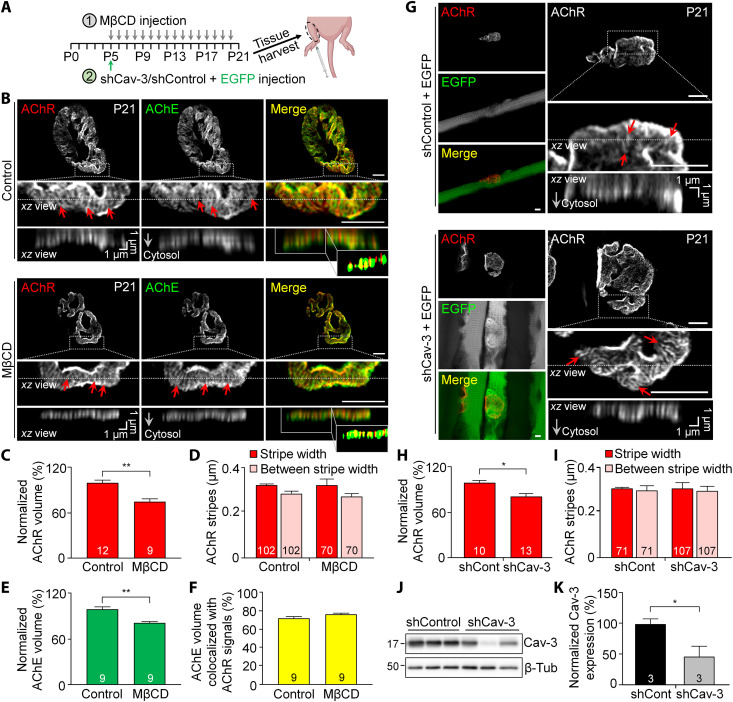
MβCD treatment or caveolin-3 knockdown inhibits the postnatal development of junctional folds at mouse NMJs in vivo. (**A**) Experimental timeline for MβCD-mediated lipid raft disruption (gray arrows) or shRNA-mediated caveolin-3 knockdown (green arrow) in the early postnatal development of mouse hindlimb muscles. (**B**) Airyscan confocal images showing the effects of MβCD on junctional fold development in single muscle fibers isolated at P21. AChE signals mark the entire junctional fold structures. Red arrows, AChR stripes. Insets: Less AChR signals (red) detected at the troughs of AChE-labeled junctional folds (green) in the thresholded images. (**C** and **D**) Quantification showing the effects of MβCD treatment on the normalized AChR volume (C) and the width of and the average distance between AChR stripes (D) at NMJs. (**E** and **F**) Quantification showing the effects of MβCD treatment on the normalized AChE volume (E) and the percentage of AChE volume colocalized with AChR signals (F) at NMJs. (**G**) Airyscan confocal images showing the effects of caveolin-3 shRNA on the development of junctional folds in single muscle fibers isolated at P21. EGFP signals: the presence of shControl or shCav-3. Red arrows: AChR stripes. (**H** and **I**) Quantification showing the effects of shRNA-mediated caveolin-3 knockdown on the normalized AChR volume (H) and the width of and the average distance between AChR stripes (I) at NMJs. shCont, shControl. (**J** and **K**) Western blot analysis (J) and quantification (K) showing the effective knockdown of endogenous caveolin-3 expression in the hindlimb muscles by adenovirus-mediated delivery of shCav-3. β-Tubulin (β-Tub) was used as the loading control. Scale bars, 5 μm (unless stated otherwise). Data are means ± SEM. The numbers indicated in the bar regions represent the total numbers of NMJs (C), (E), (F), and (H) and AChR stripes (D) and (I) measured from three animals per experimental group. *P ≤ 0.05 and **P ≤ 0.01 (Student’s *t* test).

It is well known that the structural maturation of mouse NMJs occurs during the first three postnatal weeks to enhance the efficacy of neuromuscular transmission, as hallmarked by the progressive development of postsynaptic junctional folds (*30*, *31*). To examine the requirement of lipid raft integrity and caveolin-3 expression for NMJ development *in vivo*, we examined the effects of MβCD treatment or short hairpin (shRNA)–mediated caveolin-3 knockdown on postsynaptic junctional fold development in postnatal mouse models. Specifically, we performed subcutaneous injections of MβCD daily from postnatal day 5 (P5) to P20 or a single intramuscular injection of adenovirus encoding enhanced GFP (EGFP) and control shRNA (shControl) or caveolin-3 shRNA (shCav-3) at P5, into the hindlimb muscles (Fig. 7A). By analyzing the topological features of the postsynaptic apparatus in single muscle fibers dissected from the injected muscles at P21, we found that MβCD treatment significantly inhibited postsynaptic junctional fold development, as reflected by the reduced length of AChR-enriched junctional folds in orthogonal projection images (Fig. 7B) and reduced normalized AChR volume quantitatively (Fig. 7C). In addition, we adopted the previously described quantitative analysis methods to measure AChR stripe width and the average distance between AChR stripes, which are correlated with the spatial organization of AChR molecules at the fold crests and the average width of fold crests, respectively, as determined from electron microscopy data (*6*). There was no significant change in either the stripe width or between stripe widths by MβCD treatment (Fig. 7D). By examining the localization of AChE using fluorescent fasciculin that marks the entire junctional folds (*22*, *32*), we further confirmed that MβCD treatment significantly reduced the dimension of junctional folds (Fig. 7E), rather than altering the spatial enrichment of AChRs at junctional folds (Fig. 7F). On the other hand, similar observations and quantitative results were obtained by examining the AChR-enriched junctional folds in hindlimb muscles with adenovirus-mediated delivery of shCav-3 (Fig. 7, G to I). The knockdown efficiency of endogenous caveolin-3 expression by shCav-3 was confirmed by Western blot analyses (Fig. 7, J and K). These data suggested that lipid raft integrity and precisely controlled caveolin-3 expression are required for the progressive elongation of junctional folds but do not affect the density of existing junctional folds or the spatial organization of AChR molecules at the fold crests. Together, these results provide a solid validation of our key in vitro findings for understanding the essential roles of lipid rafts and caveolin-3 in regulating the structural maturation of postsynaptic apparatus during the postnatal development of mammalian NMJs in vivo.

## DISCUSSION

Using super-resolution live-cell imaging approaches, this study first made an unexpected observation showing the presence of membrane infoldings, mirroring the junctional folds of mature NMJs, at ECM-induced aneural AChR clusters in cultured *Xenopus* muscle cells without motor nerve innervation. This is consistent with previous studies showing that nerve-independent topologically complex AChR clusters in muscle cells cultured on ECM-coated substrates undergo structural maturation, as reflected by the progressive transformation from an oval plaque into a pretzel-shaped array of branches (*10*). The present study further revealed that membrane infoldings were gradually developed at aneural AChR clusters in cultured muscles (fig. S1), which exhibited the spatial segregation of postsynaptic proteins as seen during the early postnatal maturation of vertebrate NMJs in vivo, further indicating that the topography of membrane infoldings, one of the hallmarks of postsynaptic apparatus maturation, is likely mediated by nerve-independent mechanisms.

While the formation of these nerve-independent membrane infoldings required the presence of several ubiquitously expressed ECM proteins, ECM remodeling was also required for the maintenance and elongation of membrane infoldings (Figs. 1, E and I, and 2, F and G). Within aneural AChR clusters, AChR-poor perforations contain dynamic, adhesive organelles called PLSs, which participate in the topological maturation of AChR clusters (*13*). Our recent studies demonstrated that PLSs direct the vesicular trafficking and surface insertion of membrane-type 1 MMP (MT1-MMP) to clear the extracellular space via local degradation of ubiquitously expressed ECM proteins, which, in turn, facilitates localized deposition of nerve-derived agrin to induce synaptic differentiation in both presynaptic spinal neurons and postsynaptic muscle cells (*20*, *33*–*35*). This study further showed that membrane infoldings were preferentially found at the interface of fluorescent gelatin–coated and fluorescent gelatin–degraded substratum near the perforations of aneural AChR clusters, and inhibition of MMP activity by BB-94 treatment significantly inhibited the elongation and maintenance of membrane infoldings (Fig. 2, F and G). These results suggested that MT1-MMP–mediated local ECM degradation may modulate ECM-mediated integrin signaling and/or matrix rigidity for the development of membrane infoldings.

**Fig. 8.**
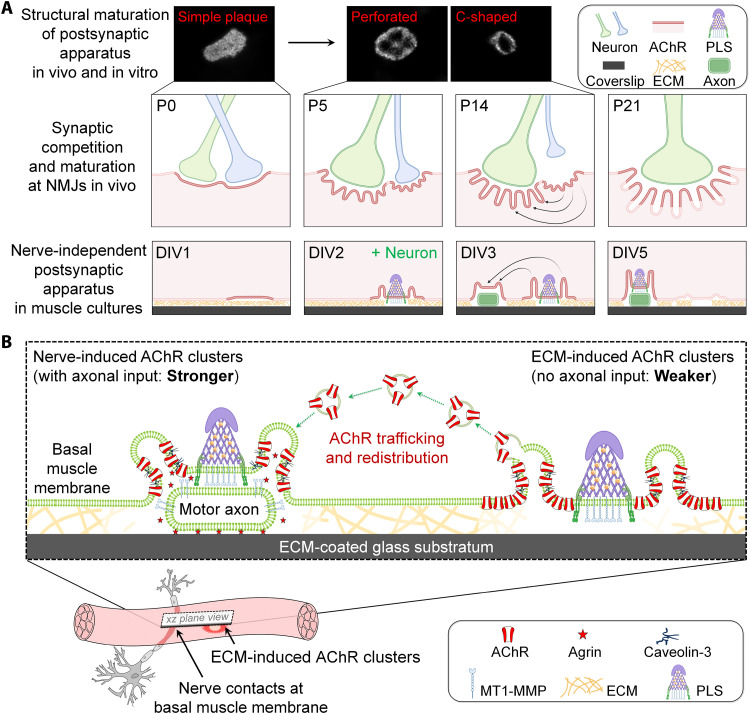
Proposed roles of membrane folds in regulating AChR trafficking and redistribution during the maturation of topologically complex postsynaptic apparatus in muscle cultures and at NMJs in vivo. (**A**) Topological changes of the postsynaptic apparatus from simple oval plaques to more elaborated perforated and C-shaped are observed in both ECM-induced AChR clusters in muscle cultures and mouse NMJs during postnatal development. Within the first three postnatal weeks (P0 to P21) in mice, the elimination of inactive axonal inputs occurs at the polyneuronally innervated muscle fibers, leading to singly innervated muscle fibers. Meanwhile, the structural maturation of postsynaptic apparatus occurs at active NMJs, as evidenced by the formation of junctional folds. In this study, we observed that membrane infoldings are progressively formed at ECM-induced AChR clusters in the basal membrane of cultured muscle cells during the first 5 days in vitro (DIV). Upon nerve innervation, AChRs are redistributed from aneural clusters to nascent nerve-muscle contact sites through intracellular trafficking of AChR vesicles derived from the membrane infoldings. This process mirrors the redistribution of postsynaptic proteins from inactive to active NMJs during synapse elimination and maturation in postnatal development of mammalian NMJs. (**B**) In *Xenopus* nerve-muscle co-cultures, we found that lipid rafts and caveolin-3 play a crucial role in regulating extensive membrane infolding formation at PLS-localized AChR-poor perforations of aneural and nerve-induced AChR clusters. Similar to the mature NMJs in vivo, vesicular trafficking of AChR molecules is observed at AChR clusters in muscle cultures, where synaptogenic stimulation may hijack the trafficking of AChR vesicles to promote the assembly of nerve-induced synaptic AChR clusters (dashed arrows). Together, this study provides compelling evidence to support the long-standing hypothesis that topological maturation of nerve-independent ECM-induced aneural AChR clusters in muscle cultures mirrors that of the postsynaptic apparatus at mature mammalian NMJs in vivo.

During early postnatal development in vivo (Fig. 8A), synapse elimination occurs at inactive NMJs, where the withdrawal of all but one motor axons can be observed in living animals (*36*). Meanwhile, structural maturation occurs at active NMJs as reflected by the progressive development of junctional folds with spatially segregated postsynaptic proteins (with AChRs clustered at the crest regions and VGSCs concentrated at the trough regions) at topologically complex postsynaptic apparatus from P0 to P21 (*7*, *37*, *38*). In contrast, AChE is known to be concentrated along the entire junctional folds (*22*, *32*). Functionally, junctional folds are believed to serve as an amplifier of neurotransmitter-induced membrane depolarization (via AChR clusters at the crests) for the initiation of action potentials (via VGSCs at the troughs) (*39*). However, immunostaining of several trough markers (using anti–pan Na_v_, anti-Na_v_1.4, and anti–ankyrin-G antibodies) showed no specific spatial enrichment of signals at aneural AChR clusters (fig. S6), indicating that the specificity of those antibodies tested is not suitable for *Xenopus* and C2C12 muscle cultures or the abundance of endogenous proteins clustered at the trough regions is too low to be detected. Nevertheless, our time-lapse super-resolution imaging experiments revealed a gradual redistribution of AChRs along the mGFP-labeled elongating membrane infoldings at the same aneural clusters over time (Fig. 2, A to E). Meanwhile, AChE signals remained to be highly enriched along the entire membrane infoldings at different time points (Fig. 3). Such progressive change in the spatial localization of AChR and AChE molecules along the membrane infoldings in cultured muscle cells further suggests the regulation of postsynaptic apparatus maturation via muscle-intrinsic mechanisms. Note that aneural AChR clusters can also be found in mouse diaphragm muscles during the initial stage of NMJ development at embryonic day 14.5 (E14.5) (*8*). However, these transiently present aneural AChR clusters, which are subsequently recruited to nascent postsynaptic sites by E18.5, do not develop obvious membrane infoldings. Instead, the ECM-induced AChR clusters that we observed in cultured muscle cells mimic the postsynaptic apparatus at inactive NMJs during synaptic elimination in postnatal development (Fig. 8B). We speculate that spatially enriched PLSs at ECM-induced aneural and synaptic AChR clusters in muscle cultures accelerate the structural maturation of postsynaptic specializations, making them resemble the topological features and molecular composition of the postsynaptic apparatus that is normally observed at mature NMJs in vivo. The formation of nerve-induced synaptic AChR clusters coupled with the dispersal of ECM-induced aneural AChR clusters in muscle cultures could therefore potentially be used to study the molecular regulation of AChR redistribution from inactive to active NMJs during synaptic elimination and maturation in postnatal development in vivo. Nevertheless, the present study reports a previously unknown observation that membrane infoldings with spatially segregated postsynaptic proteins, one of the hallmarks of postsynaptic apparatus maturation, can be developed via nerve-independent mechanisms.

Lipid rafts are known to serve as a signaling platform for NMJ formation through intracellular trafficking of AChRs and rapsyn (*14*, *15*), and the phase separation of rapsyn can form membraneless condensates to recruit different molecular components during NMJ development (*40*). While lipid raft disruption causes drastic effects on AChR clustering, whether lipid rafts are involved in the formation of membrane infoldings remains unclear. In this study, we found that MβCD treatment for acute depletion of membrane cholesterol significantly inhibited the formation of membrane infoldings and the elongation of the existing ones at aneural AChR clusters (Fig. 4, B and C). Because MβCD is also known to affect various membrane processes, such as clathrin-mediated and caveolar endocytic pathways, endosomal trafficking, and phosphatidylinositol 4,5-bisphosphate localization (*41*, *42*), we alternatively used another pharmacological agent, filipin III, which binds to and sequesters membrane cholesterol and disrupts lipid rafts differently from MβCD (*43*). Similar to the effects of MβCD treatment, filipin III also significantly reduced the volume of membrane infoldings associated with aneural AChR clusters (fig. S7). Caveolin-3 is the muscle-specific caveolin isoform that serves as the principal component of a subset of rafts, called caveolae. At NMJs, caveolin-3 is colocalized with AChR clusters and is required for AChR clustering in cultured myotubes through MuSK-Rac1 signaling (*17*, *18*). However, whether caveolin-3 is involved in membrane infolding formation remains unexplored. Consistent with the effects of MβCD treatment (fig. S8), we demonstrated that low caveolin-3 knockdown levels significantly inhibited membrane infolding formation at aneural AChR clusters (Fig. 4, I to K), which subsequently attenuated the formation of synaptic AChR clusters induced by spinal neurons (Fig. 5, D to F) or agrin stimulation (Fig. 6, A to C). These findings therefore reveal a previously unappreciated role of lipid rafts and caveolin-3 in membrane infolding formation, different from their known functions in AChR clustering. Although caveolin-3 is involved in different cellular events (such as the early development of T-tubules in muscles) (*27*, *44*), no observable differences were found in CellMask-labeled T-tubule structures between wild-type, control MO, and low caveolin-3 MO muscle cells (fig. S9). This indicated that our observed defects in membrane infolding formation by low caveolin-3 knockdown are unlikely contributed by severe and general impacts on muscle cell health. In caveolin-3 null mice, they exhibit abnormal NMJ activity that is contributed by defects in AChR clustering (*17*). Because the structural defects in junctional folds, if any, in caveolin-3 knockout mice cannot be ruled out by the secondary effects of AChR clustering inhibition, the use of caveolin-3 knockout animal models to study membrane infolding formation is not suitable in the present study. Alternatively, we investigated the effects of shRNA-mediated caveolin-3 knockdown on the topological features of junctional folds at postnatal NMJs in wild-type mice by whole-mount analyses as previously described (*45*). Our results showed that shRNA-mediated caveolin-3 knockdown significantly inhibited the progressive elongation of AChR-enriched postsynaptic junctional folds between P5 and P21 (Fig. 7, G to K), demonstrating the involvement of caveolin-3 in junctional fold development at mammalian NMJs in vivo.

Synaptic podosomes are believed to promote the structural maturation of postsynaptic apparatus in postnatal NMJ development in mice (*13*). Given that PLSs are highly enriched at AChR clusters in *Xenopus* primary muscle cells cultured on ECM substratum (*20*), it is not surprising to see that the topological features of aneural and synaptic AChR clusters observed in this study inevitably resemble topologically complex postsynaptic apparatus at mature NMJs in vivo. Our results showed that caveolin-3 knockdown significantly reduced the number of AChR-containing vesicles at aneural AChR clusters and inhibited the redistribution of AChRs from aneural to synaptic clusters upon agrin stimulation (Fig. 6, D and E), suggesting that AChR vesicles derived from membrane infoldings at aneural clusters may be directed to the nascent postsynaptic apparatus upon synaptogenic stimulation in *Xenopus* nerve-muscle co-cultures. Upon agrin bead stimulation, we observed AChR-containing vesicles that are derived from membrane infoldings at ECM-induced aneural AChR clusters, followed by directional transport toward the local synaptogenic stimulation by agrin beads (Fig. 6G and movie S1). Vesicular trafficking of AChRs has been implicated in agrin-induced AChR transcytosis for the assembly of nascent postsynaptic specializations at developing *Xenopus* NMJs in cultures (*11*, *46*, *47*) and activity-dependent AChR recycling for the modulation of receptor density and synaptic efficacy at mature NMJs in living mice (*48*, *49*). We therefore hypothesize that membrane infoldings associated with AChR clusters may serve as an endo/exocytic hub for AChR vesicular trafficking in nerve-muscle co-cultures (Fig. 8B). Consistent with our hypothesis, a recent study revealed that dynamin-2, a ubiquitously expressed guanosine triphosphatase involved in different stages of the secretory pathway, serves as a membrane scission catalyzer to facilitate synaptic vesicle recycling in presynaptic terminals and as a molecular girdle to regulate PLS maturation and actin organization in postsynaptic muscles at *Drosophila* NMJs in vivo (*50*). It is of interest to examine whether dynamin-2 also regulates the formation of AChR vesicles at membrane infoldings by mediating fission at the neck of caveolae as observed in other cell types (*51*). In sum, the *Xenopus* culture system provides a unique platform for studying how AChR and other postsynaptic components are redistributed via vesicular trafficking at junctional folds from inactive to active mammalian NMJs during naturally occurring synaptic elimination and maturation in postnatal development (Fig. 8A). With recent advances in lattice light-sheet microscopy (*52*), it would be possible to perform high-speed super-resolution imaging on living specimens with minimal phototoxicity that may allow us to provide more definite evidence for the involvement of AChR trafficking at developing NMJs in vivo in future studies.

Abnormalities of the junctional fold architecture are the most common phenotypes associated with several neuromuscular disorders in humans (*53*). In particular, changes in the expression of the caveolin-3 gene have been associated with different types of muscular dystrophies (*28*, *54*). A significant increase in the number of caveolae at the sarcolemma is associated with the over-production of caveolin-3 in patients with Duchenne muscular dystrophy (DMD) (*55*, *56*). On the other hand, mutations in the caveolin-3 gene causing the loss of caveolin-3 expression are responsible for an autosomal dominant form of limb-girdle muscular dystrophy (LGMD) (*57*). These studies indicate that the precise control of endogenous caveolin-3 expression levels is essential for maintaining normal NMJ structures and function, as well as for organizing the dystrophin-glycoprotein complex that is important for the maturation and maintenance of the postsynaptic apparatus (*58*). As membrane infoldings are developed in association with ECM-induced aneural and synaptic AChR clusters in *Xenopus* primary muscle cultures that has been used as a cell-based assay for studying the pathogenesis of myasthenia gravis (*59*), this assay could be applied for investigating the formation and maintenance of membrane infoldings at AChR clusters in human primary muscle cultures derived from DMD or LGMD patients. Collectively, the results of our study provide a better understanding of the molecular regulation of nerve-independent, caveolin-3–dependent membrane infolding formation and maintenance at the postsynaptic apparatus, which could potentially be translated to understand how junctional fold abnormalities impair synaptic functions in various neuromuscular diseases.

## MATERIALS AND METHODS

### Preparation of *Xenopus* embryonic muscle and nerve-muscle cultures

Adult *Xenopus laevis* animals were purchased from Xenopus 1. *Xenopus* oocytes were fertilized in vitro, and the embryos were raised in 10% Holtfreter’s solution [v/v; 60 mM NaCl, 0.6 mM KCl, 0.9 mM CaCl_2_, and 0.2 mM NaHCO_3_ (pH 7.4)] at 22°C (*11*). A total of 460 to 690 pg of DNA construct encoding pCAG-membrane GFP (mGFP) (Addgene, #14757; RRID: Addgene_14757) was microinjected into one blastomere of one- or two-cell stage *Xenopus* embryos with a microinjector. mGFP-expressing embryos were screened for primary culture preparation. The dorsal parts of embryos at the Nieuwkoop and Faber stages 19 to 22 were dissected as previously described (*60*). After enzymatic digestion by collagenase (Sigma-Aldrich, C98191G), myotomal tissues and neural tubes were isolated, followed by dissociation with calcium-magnesium–free solution. The dissociated cells were plated on coverslips or glass-bottom dishes coated with a mixture of cell attachment substrate, ECL (Merck Millipore, 08-100), or specific ECM proteins including laminin (Sigma-Aldrich, L2020), collagen I (Fisher Scientific, C354249), and gelatin (Sigma-Aldrich, G1393) at 10 μg/ml or poly-d-lysine (Sigma-Aldrich, P1024) at 100 μg/ml for approximately 3 hours at 37°C. Cells were maintained in culture medium containing 87% Steinberg’s solution [v/v; 60 mM NaCl, 0.67 mM KCl, 0.35 mM Ca(NO_3_)_2_, 0.83 mM MgSO_4_, 10 mM Hepes, 10% Leibovitz’s L-15 medium, 1% fetal bovine serum (v/v), 1% penicillin-streptomycin (v/v), and 1% gentamicin sulfate (v/v)]. Muscle cells were kept at 22°C for at least 48 hours to allow cell attachment and development of aneural AChR clusters before treatments, if any. To make nerve-muscle or bead-muscle co-cultures, dissociated spinal neurons or polystyrene latex beads coated with recombinant agrin (R&D Systems, 550-AG-100/CF) were added to 2-day-old muscle cultures and grown for the specified time before imaging as previously described (*11*, *61*). All experiments involving *Xenopus* frogs and embryos were carried out in accordance with the approved protocols by the Committee on the Use of Live Animals in Teaching and Research (CULATR) of The University of Hong Kong.

### Morpholino-mediated knockdown of endogenous proteins

Knockdown of endogenous proteins in *Xenopus* was achieved by embryonic injection of antisense MOs (GeneTools), which bind to the target mRNA sequence to block its protein translation. The following MO sequences were used in this study: *Xenopus* caveolin-3 MO, 5′-TTCTGCCATAACTCTTTCTTGCATC-3′ and standard control MO, 5′-CCTCTTACCTCAGTTACAATTTATA-3′. A total of 12 ng (low dose of caveolin-3 MO) or 19 ng (high dose of caveolin-3 MO) was microinjected into one blastomere of one- or two-cell stage *Xenopus* embryos. To visualize the presence of MO in the microinjected embryos, Alexa Fluor 488–conjugated (Thermo Fisher Scientific, D22910) or Alexa Fluor 546–conjugated dextran (Thermo Fisher Scientific, D22911) was coinjected as a cell lineage tracer. The effectiveness of MO-mediated knockdown of endogenous proteins was validated by Western blot analyses and immunostaining.

### Preparation of whole-mount mouse skeletal muscle fibers

Wild-type mice were euthanized by cervical dislocation. The extensor digitorum longus (EDL) and tibialis anterior muscles were dissected and fixed with 4% paraformaldehyde for 30 min. AChRs were labeled with 0.1 μM tetramethylrhodamine (Rh)–conjugated BTX (Thermo Fisher Scientific, T1175) overnight at 4°C in 4% Triton X-100. Muscle fibers were then mounted on glass slides with the anti-bleaching reagent Fluoromount-G with 4′,6-diamidino-2-phenylindole (Thermo Fisher Scientific, 00-4959-52) for later observation.

For experiments studying the effects of shRNA-mediated caveolin-3 knockdown on junctional fold development at NMJs in vivo, P5 C57BL/6J male mice (Centre for Comparative Medicine Research, The University of Hong Kong) were briefly anesthetized with isoflurane (3 to 4% for induction and 1.5% for maintenance). Then, the right tibialis anterior muscles were intramuscularly injected with 7 μl of adenovirus (OBiO Technology) to mediate the delivery of plasmids encoding EGFP and control shRNA (shControl) or Cav-3 shRNA (shCav-3) at 2.17 × 10^8^ plaque-forming units per site. The following shRNA sequences were used in this study (target sequences are underlined): shCav-3, 5′-CCGGGCTTCGACGGTGTATGGAATTCAAGAGATTCCATACACCGTCGAAGCTTTTTTG-3′ and shControl, 5′-CCGGTTCTCCGAACGTGTCACGTTTCAAGAGAACGTGACACGTTCGGAGAATTTTTTG-3′. At P21, the injected muscles were identified by the expression of EGFP and then dissected from euthanized animals. Single muscle fibers were isolated for whole-mount analyses. All experiments were carried out in accordance with the approved CULATR protocol.

### C2C12 myotube culture

C2C12 cells (American Type Culture Collection, CRL-1772; RRID: CVCL-0188) were cultured as described previously (*12*). For microscopy, myoblasts were plated on glass-bottom dishes (IBIDI, 81158) coated with laminin 111 (Sigma-Aldrich, L2020). Four days after differentiation induction, cells were stained with CellMask Green Plasma Membrane Stain (5 μg/ml; Thermo Fisher Scientific, C37608) for 7 min and imaged live on a Zeiss Cell Observer SD confocal microscope (Carl Zeiss) using a 63× oil objective [numerical aperture (NA) 1.40] and a QImaging ROLERA EM-C2 electron-multiplying charge-coupled device (EMCCD) camera. CellMask Green Plasma Membrane Stain was excited with a 488-nm laser line, and the respective emission was collected with an FE01-520/35 emission filter.

### Fluorescent gelatin degradation assay

Glass coverslips (Fisher Scientific, 12-545-82) were coated with Oregon Green 488-gelatin (1 mg/ml; Thermo Fisher Scientific, G13186) for 10 min, followed by cross-linking with 0.5% glutaraldehyde (Sigma-Aldrich, G5882) in phosphate-buffered saline (PBS) for 15 min (*20*). After that, gelatin-coated coverslips were treated with NaBH_4_ (5 mg/ml; Sigma-Aldrich, 452882) for 3 min. Dissociated cells were plated on fluorescent gelatin–coated coverslips and maintained for 3 days before imaging.

### Pharmacological treatment

For experiments studying the effects of MMP inhibition, 5 μM BB-94 (ApexBio, A2577) was added to the culture medium before muscle cell plating for pre-treatment conditions or to the 3-day-old muscle cultures immediately after taking the first time point images for post-treatment conditions. For experiments studying the effects of lipid raft disruption, 2 mM MβCD (Sigma-Aldrich, C4555) or 3.82 μM filipin III (Sigma-Aldrich, F4767) was added to the culture medium before muscle cell plating for pre-treatment conditions or to the 3-day-old muscle cultures immediately after imaging at the first time point for post-treatment conditions.

For experiments studying the effects of lipid raft disruption on junctional fold formation at NMJs in vivo, P5 C57BL/6J male mice were briefly anesthetized with 3 to 4% isoflurane, and MβCD was subcutaneously injected into the right hindlimbs at 10 mg per kilogram of their body weight. The uninjected left hindlimbs of the same animals were used as the control, and the procedure was repeated daily until the mice reached P21 for single muscle fiber dissection and whole-mount analyses. All experiments were carried out in accordance with the approved CULATR protocol.

### Endogenous agrin track assay

Following procedures previously described in (*20*), we plated dissociated *Xenopus* spinal neurons onto ECL-coated coverslips and cultured for 1.5 days. Spinal neurons were digested and removed by incubating with 1% Triton X-100 in PBS for 4 min, followed by extensive washing with PBS at least 5 times. Dissociated muscle cells were then plated onto the coverslips and grown for another 4 days. The location of agrin tracks was visualized by agrin immunostaining (1:100; DSHB, 6D2; RRID: AB_528071) and then confirmed if neurites were not found in the phase-contrast images.

### Labeling of different AChR pools in cultured muscle cells

Following procedures previously described to differentially label surface and internal AChRs (*11*), cells were first fixed with 4% paraformaldehyde in PBS for 15 min. Surface AChRs were labeled with 0.1 μM Rh-BTX for 45 min, followed by incubation with a saturating dose (6 μM) of unconjugated BTX for 30 min. After extensive washing, cells were permeabilized with 0.5% Triton X-100, and internal AChRs were labeled with 0.1 μM Alexa Fluor 488–conjugated BTX for 45 min. Coverslips were then mounted on glass slides with the anti-bleaching reagent Fluoromount-G (Thermo Fisher Scientific, 00-4958-02) for later observation.

### Live cell staining

To label AChR clusters, live muscle cells on coverslips or glass-bottom dishes were stained with 0.1 μM Rh-BTX for 45 min. To label the cell membrane, cultures were stained with CellMask deep red plasma membrane stain (5 μg/ml; Thermo Fisher Scientific, C10046) for 7 min. To visualize lipid rafts, cultures were stained with 1 μg/ml Alexa Fluor 488-conjugated CTX (Thermo Fisher Scientific, C34775) for 7 min. To label AChE, cultures were stained with 150 nM Alexa Fluor 488–conjugated fasciculin 2 (Alomone, F-225) for 75 min.

### Cell fixation and immunostaining

Cultured wild-type cells were fixed with 4% paraformaldehyde for 15 min, and cultured mGFP-expressing cells were fixed with 1% paraformaldehyde for 20 min. Fixed cells were then permeabilized with 0.1% Triton X-100 for 15 min. After extensive washing with PBS at least three times, they were incubated with 2% bovine serum albumin (Sigma-Aldrich, A9418) at 4°C overnight. Cells were incubated with caveolin-3 antibody (1:300; Covalab, 74755; RRID: AB_2088281), pan Na_v_ antibody (1:200; Alomone, ASC-003; RRID: AB_2040204), Na_v_1.4 antibody (1:200; Alomone, ASC-020; RRID: AB_2040009), or ankyrin-G antibody (1:100; Thermo Fisher Scientific, 33-8800; RRID: AB_2533145 or 1:100; DSHB, N106/36; RRID: AB_2921666) at room temperature for 2 hours, followed by fluorophore-conjugated secondary antibodies (1:400; Thermo Fisher Scientific) for 45 min. Coverslips were then mounted on glass slides with Fluoromount-G for later observation.

### Western blot analysis

Nieuwkoop and Faber stage 19 to 22 *Xenopus* embryos were homogenized in radioimmunoprecipitation assay buffer in the presence of protease inhibitor cocktail and EDTA, followed by incubation on ice for 5 min. After high-speed centrifugation (15,000*g*), the supernatant was collected for protein concentration measurement using a BCA protein assay kit (Thermo Fisher Scientific, 23227). Protein lysates (30 μg) were used for protein separation by SDS–polyacrylamide gel electrophoresis (SDS-PAGE) and then blotted onto polyvinylidene difluoride membranes (Bio-Rad, 1620174). After blocking with 5% nonfat milk in tris-buffered saline with tween 20 (TBST), the blots were probed with the following primary antibodies: caveolin-3 (1:1500) or β-tubulin (1:1000; DSHB, E7; RRID: AB_528499) at 4°C overnight. After extensive washing with TBST, the blots were probed with horseradish peroxidase–conjugated secondary antibodies. Signals were detected after incubation with ECL Western blotting substrate (Thermo Fisher Scientific, 32106) using a ChemiDoc XRS+ System (Bio-Rad).

In the dissected tibialis anterior muscles from P21 C57BL/6J male mice, one-third of the muscle samples were used for Western blot analyses. The muscle samples were flash-frozen in liquid nitrogen, followed by homogenization in lysis buffer (50 mM tris, 40 mM NaCl, 1.5 mM Na_3_VO_4_, 50 mM NaF, 10 mM Na_4_P_2_O_7_, 10 mM sodium β-glycerol phosphate, and 0.5% Triton X-100) in the presence of protease inhibitor cocktail and EDTA. The supernatant was collected after high-speed centrifugation (20,000*g*) for 10 min at 4°C. The protein concentration was measured by the Bradford protein assay (Bio-Rad, 5000006). Protein lysates (5 μg) were separated by SDS-PAGE and then blotted onto nitrocellulose membranes (Bio-Rad, 1620112). After blocking with 5% nonfat milk in TBST, the blots were probed with the primary antibodies: caveolin-3 (1:2000) or β-tubulin (1:1000) at 4°C overnight. After extensive washing with TBST, the blots were probed with horseradish peroxidase–conjugated secondary antibodies. Signals were detected after incubation with ECL Western blotting substrate (UltraScience, CCH345) using a G:BOX Chemi XRQ imager (Syngene).

### Fluorescence microscopy

Wide-field fluorescence imaging was performed on inverted microscopes (IX83, Olympus) using an oil-immersion PlanApo 60× NA 1.42 objective lens. Digital still images were captured by ORCA-Flash4.0 LT+ digital CMOS camera (Hamamatsu) through the software MicroManager (Open Imaging) (*62*). For imaging of membrane infoldings, multiple *z*-stack images were captured on a confocal microscope (LSM 880 with Airyscan, Carl Zeiss) using an oil-immersion 63× NA 1.4 DIC objective lens. Images were captured with 32-channel GaAsp photomultiplier modules using ZEN 2.3 software (Carl Zeiss). All confocal *z*-stack images were subjected to Airyscan processing. For imaging and tracking of AChR vesicles, high-speed time-lapse images were captured at 200 ms per frame on live-SR super-resolution module on an iLas3 Ring-TIRF system (Gataca Systems). All acquisition settings (i.e., laser power and gain) were kept the same for different experimental groups in the same experiment. All acquired images were analyzed by ImageJ (NIH) or Imaris (Bitplane).

### Data analysis

To quantify the percentage of aneural AChR top and bottom clusters, muscle cells were first identified in the phase-contrast images, and then, the presence of top or bottom aneural AChR clusters was scored in the fluorescence images.

To quantify the percentage of nerve/bead-induced AChR clusters, nerve contacts with the basal membrane of muscle cells or agrin bead contacts with the top membrane of muscle cells were first identified in the phase-contrast images, and then, the presence of nerve/bead-induced AChR clusters was scored. To quantify the percentage of aneural AChR clusters with infoldings, muscle cells with bottom aneural AChR clusters were first identified, and then, the presence of membrane infoldings was scored.

To quantify the percentage of the perforated area of aneural AChR clusters, separate regions of interest (ROIs) were created for measuring the AChR-poor and entire AChR clusters. The area of the perforated AChR-poor region was then normalized against that of the entire AChR clusters. To quantify the normalized intensity of bead-induced AChR clusters, the fluorescence intensity of AChRs at the bead-muscle contacts was first measured in all experimental groups and then normalized to the average AChR intensity in the control group of the same experiment. To quantify the fluorescence intensity of nerve-induced AChR clusters per unit length of nerve-muscle contact, the integrated fluorescence value of AChR signals was first measured and then normalized against the length of nerve-muscle contacts as measured in the phase-contrast images.

To quantify the number of internal AChR puncta per unit area of AChR clusters, the number of internal AChR puncta was manually counted and then normalized against the area of aneural AChR clusters in the same bead-contacted muscle cell. To quantify the percentage change in mGFP fluorescence intensity in the same AChR clusters over 3 days, mGFP signals at different time points were measured and then normalized against that at the first time point taken on day 3 in culture.

To quantify the volume of membrane infoldings and the colocalization volume of AChRs and membrane infoldings, the volumes of membrane infoldings, AChR, and their colocalization were first measured using the surface rendered images generated in Imaris. The volume of membrane infoldings was then normalized against the AChR area of the same clusters to account for the variation in the size of different AChR clusters. To quantify the fold change in membrane infolding volume or colocalization volume, the volumes measured after 1 day were normalized against the measured values on day 0. To quantify the volume of AChR clusters and AChE localization at mouse NMJs in vivo, AChR or AChE volume was normalized against their area in the maximal projection images.

To quantify the width of and distance between the AChR stripes at mouse NMJs in vivo, line profiles of AChR intensity were generated using lines drawn perpendicular to the AChR stripes near the midline. The width of AChR stripes and the distance between two AChR stripes were measured at the 50% AChR intensity level.

To quantify the fluorescence intensity of caveolin-3 at aneural AChR clusters, an ROI of the AChR clusters was first created and then applied to the caveolin-3 fluorescence images for the measurement. The same ROI was also used to measure the in-cell fluorescence background. The fluorescence intensity of caveolin-3 at the AChR clusters was then normalized to the background in the same cell.

In all figures, the mean and SEM values are shown in the graphs, unless otherwise specified. The numbers of biological replications and the statistical tests applied are specified in the figure legends.

## Notes

View/request a protocol for this paper from *Bio-protocol*.
