# Supplementary material for: Nerve-independent formation of membrane infoldings at topologically complex postsynaptic apparatus by caveolin-3
Source: Sci Adv. 2023 Jun 16;9(24):eadg0183. doi: 10.1126/sciadv.adg0183 (PMC10275590; doi:10.1126/sciadv.adg0183)
Supplement: Supplementary file 1 — Figs. S1 to S9 Legend for movie S1 [file sciadv.adg0183_sm.pdf]

Science Advances
AAAS

Supplementary Materials for

Nerve-independent formation of membrane infoldings at topologically complex postsynaptic apparatus by caveolin-3

Hui-Lam Rachel Kwan *et al.*

Corresponding author: Chi Wai Lee, chiwai.lee@hku.hk

Sci. Adv. **9**, eadg0183 (2023)
DOI: 10.1126/sciadv.adg0183

The PDF file includes:

Figs. S1 to S9
Legend for movie S1

Other Supplementary Material for this manuscript includes the following:

Movie S1

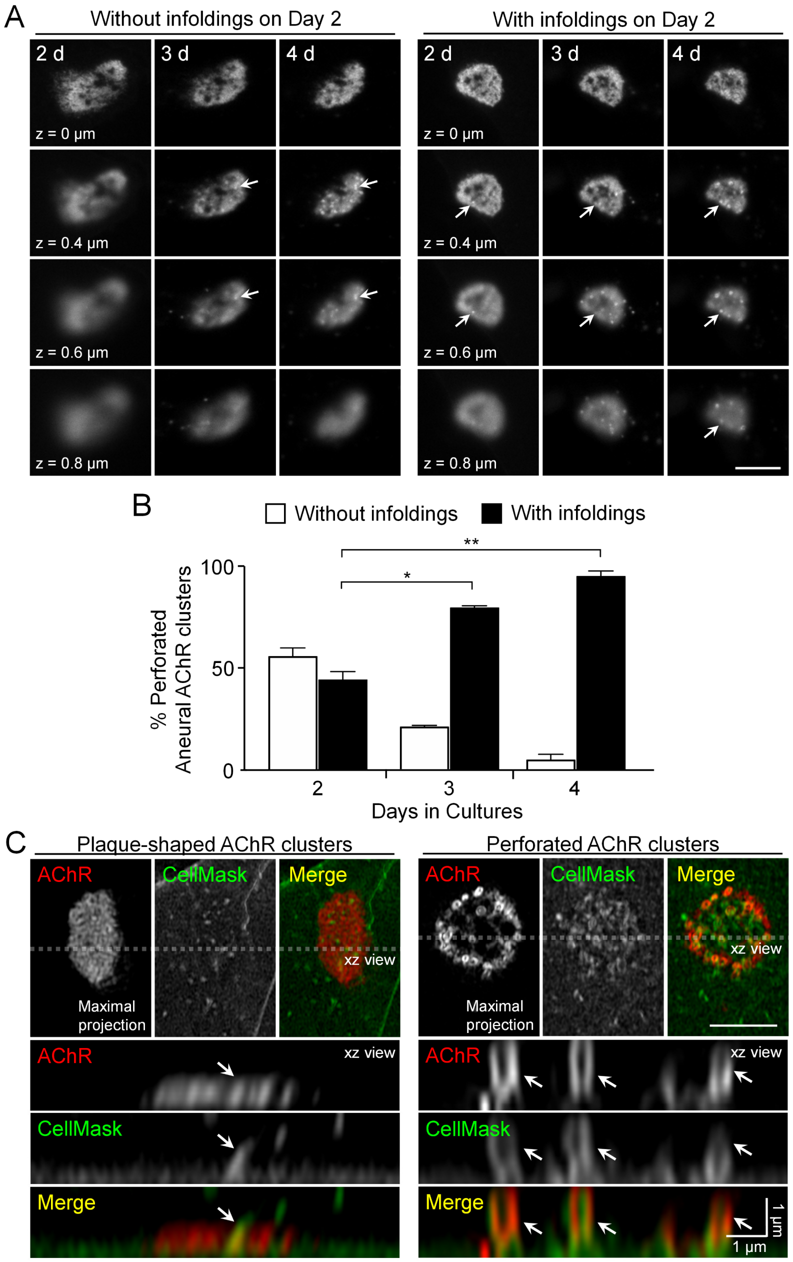

Fig. S1.

Membrane infoldings are progressively developed at aneural AChR clusters in cultured *Xenopus* muscles over time.

(**A**) Different focal planes of wide-field epifluorescence z-stack images (taken at 0.2 µm intervals) showing the progressive formation and maintenance of membrane infoldings (arrows) within the same aneural AChR clusters in muscle cells cultured for 2 to 4 days. Left panels indicate an example capturing the formation of membrane infoldings in aneural AChR clusters initially with no membrane infoldings on day 2. Right panels indicate an example showing the increase in the number and length of membrane infoldings in muscle cells over the first 4 days in cultures.

(**B**) Quantification showing the significant increase in the percentage of perforated AChR clusters with membrane infoldings in muscle cells cultured for 2 to 4 days. n = 57 muscle cells from 3 independent experiments.

(**C**) Airyscan confocal images showing that membrane infoldings are frequently found in association with topologically complex perforated AChR clusters (right panels), compared to the simple oval plaque-shaped AChR clusters (left panels) in cultured *Xenopus* primary muscle cells. Arrows indicate the spatial colocalization of AChR and membrane infoldings in the orthogonal views.

Scale bars represent 5 µm, unless stated otherwise. Data are mean ± SEM. *, ** represent $p \leq 0.05$, and 0.01 (One-way ANOVA with Dunnett's multiple comparisons test).

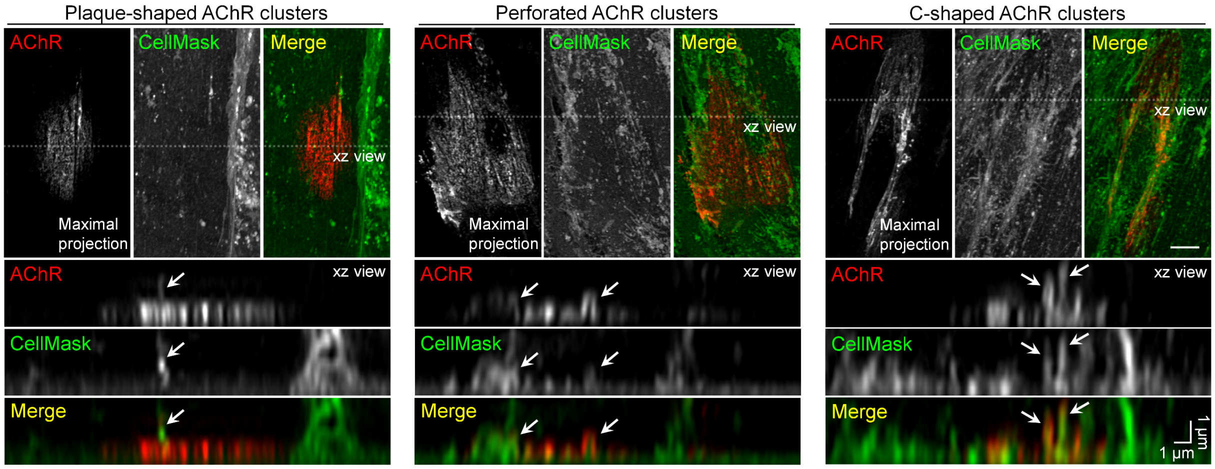

Fig. S2.

Membrane infoldings are associated with topologically complex aneural AChR clusters in C2C12 myotubes.

Representative Airyscan confocal images showing that membrane infoldings are frequently found in association with topologically complex perforated (middle panels) and C-shaped AChR clusters (right panels), compared to the simple oval plaque-shaped AChR clusters (left panels) in C2C12 myotubes cultured on laminin-coated substratum. Arrows indicate the spatial colocalization of AChR and membrane infoldings in the orthogonal views.

Scale bar represents 5 µm, unless stated otherwise.

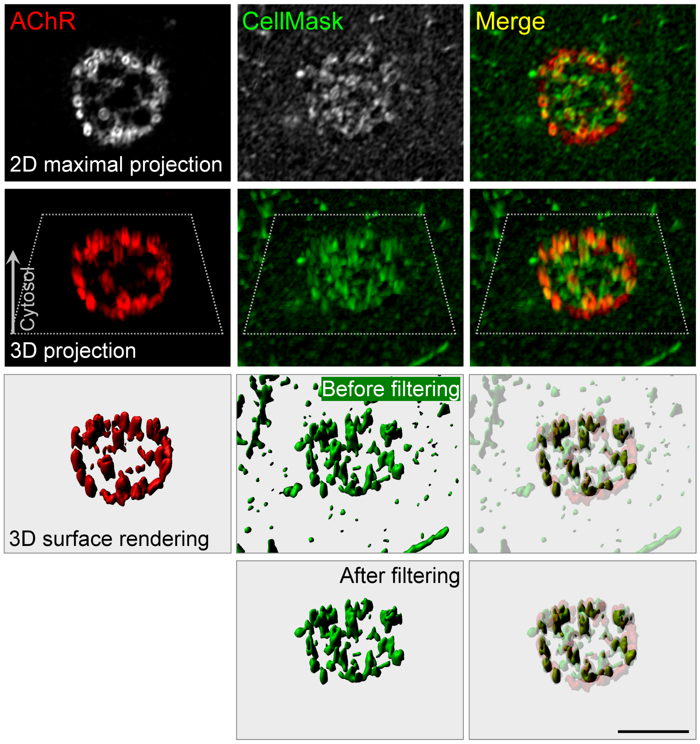

Fig. S3.

The presence of membrane infoldings at aneural AChR clusters is highlighted by different image processing approaches.

Representative images showing different image processing approaches that were used in this study to highlight the presence of membrane infoldings at aneural AChR clusters in Airyscan confocal super-resolution images. Maximal projection images were used to display the brightest pixels in the entire z-stack images in a single 2D image. 3D reconstruction and surface rendered images were generated by processing the confocal z-stack images with volume render and surface display parameters. For clarity, a manual filtering process was performed to filter out membrane signals at non-AChR cluster regions.

Scale bar represents 5 µm.

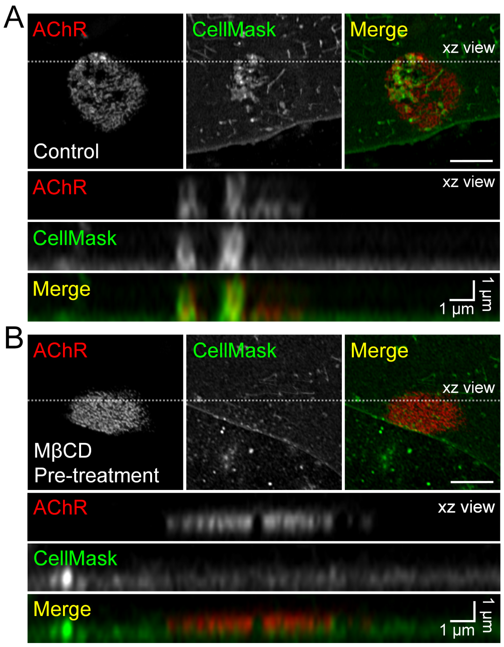

Fig. S4.

MβCD pre-treatment inhibits the formation of topologically complex aneural AChR clusters and their associated membrane infoldings.

Maximal projection of confocal z-stack images showing the differences in topological features of aneural AChR clusters and their associated membrane infoldings in control (**A**) and MβCD pre-treated (**B**) muscle cells. In the MβCD pre-treatment condition, all aneural AChR clusters exhibited simple oval plaque-shaped structures with no membrane infoldings.

Scale bars represent 5 µm, unless stated otherwise.

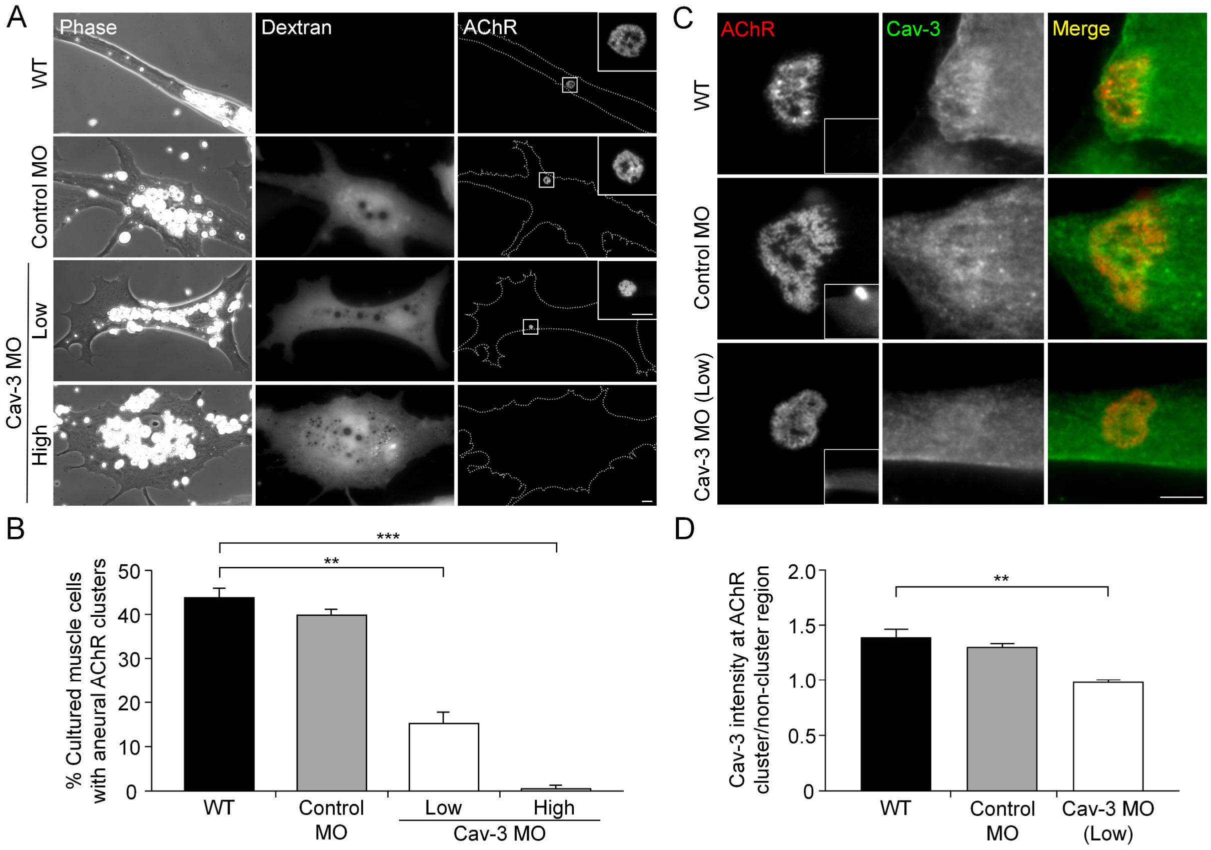

Fig. S5.

Caveolin-3 knockdown inhibits aneural AChR cluster formation.

(**A**) Representative images showing the dose-dependent effects of caveolin-3 MO on aneural AChR cluster formation in cultured muscle cells. Dotted lines outline the periphery of muscle cells. Insets show the magnified view of AChR clusters.

(**B**) Quantification showing the dose-dependent inhibition of aneural AChR cluster formation by caveolin-3 MO. n = 150 muscle cells in each condition from 3 independent experiments.

(**C**) Representative images showing the reduced localization of endogenous caveolin-3 proteins associated with aneural AChR clusters by caveolin-3 MO. Insets show the fluorescent dextran signals, indicating the presence of MO.

(**D**) Quantification showing the effects of caveolin-3 MO on the endogenous caveolin-3 intensity at aneural AChR clusters against non-cluster regions. n = 37 (WT), 23 (Control MO), and 16 (Cav-3 MO (Low)) muscle cells from 3 independent experiments.

Scale bars represent 5 µm. Data are mean ± SEM. **, *** represent $p \leq 0.01$, and 0.001 (One-way ANOVA with Dunnett's multiple comparisons test).

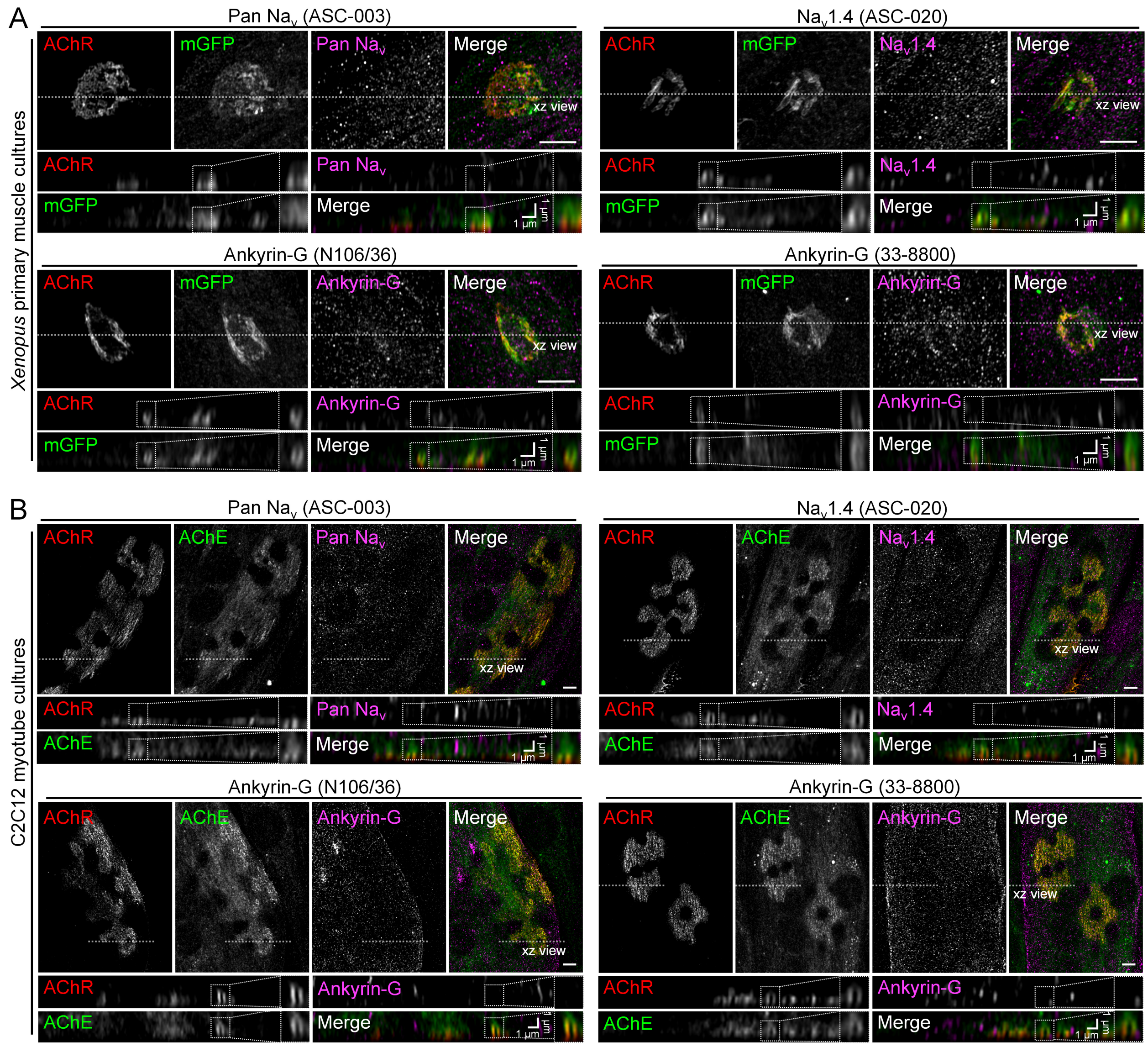

Fig. S6.

Immunostaining using selected antibodies against several trough markers shows no spatially enriched signals in the membrane infoldings at aneural AChR clusters.

Representative images showing no spatial enrichment of several trough markers (pan Na$_V$, Na$_V$1.4, and ankyrin-G) at aneural AChR clusters and their associated membrane infoldings in *Xenopus* muscle (**A**) and C2C12 myotube cultures (**B**). The catalog number of antibodies was indicated in the brackets. For clarity, insets show the magnified view (1.5X) of single membrane infolding structures.

Scale bars represent 5 µm, unless stated otherwise.

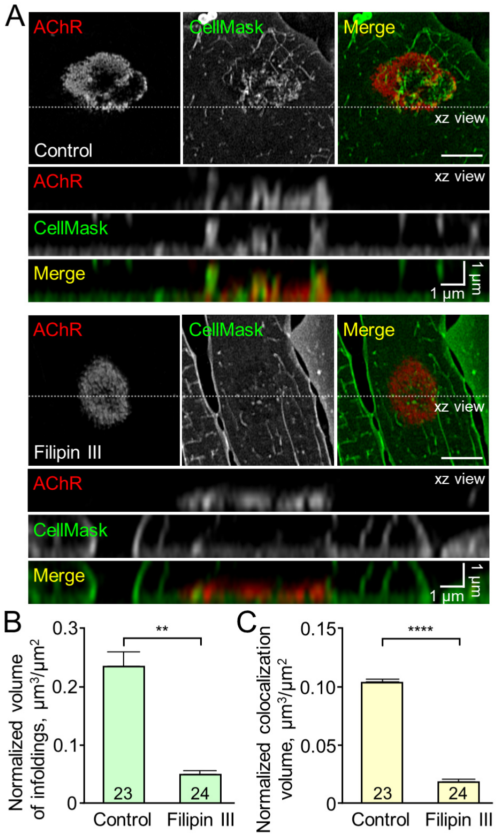

Fig. S7.

Filipin treatment inhibits the formation of membrane infoldings at aneural AChR clusters.

(**A**) Representative images showing the inhibitory effects of filipin III on the formation of membrane infoldings in aneural AChR clusters.

(**B-C**) Quantification showing the normalized volume of membrane infoldings (**B**) and colocalization volume of AChRs and membrane infoldings (**C**) per unit area of aneural clusters.

Scale bars represent 5 µm, unless stated otherwise. Data are mean ± SEM. The numbers indicated in the bar regions represent the total numbers of muscle cells quantified from 3 independent experiments. **, **** represent $p \leq 0.01$, and 0.0001, (Student's t-test).

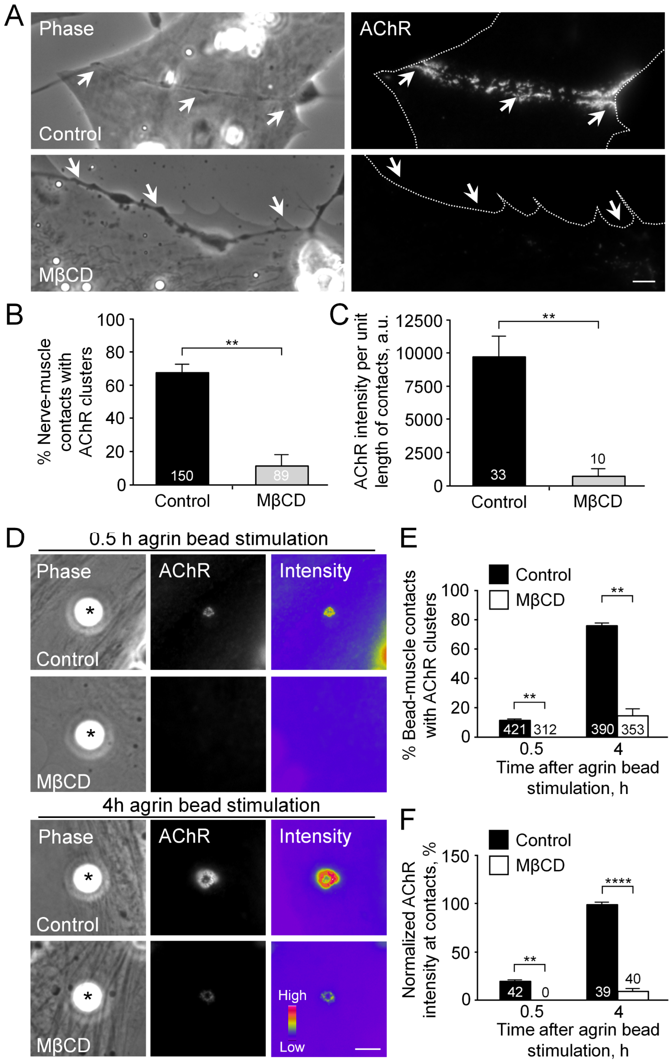

Fig. S8.

MβCD pre-treatment inhibits the formation of nerve- or agrin bead-induced synaptic AChR clusters and their associated membrane infoldings.

(**A**) Representative images showing the significant inhibition of nerve-induced AChR clustering by MβCD pre-treatment. Arrows indicate sites of nerve-muscle contacts.

(**B**-**C**) Quantification showing the inhibitory effects of MβCD pre-treatment on the percentage of nerve-muscle contacts with AChR clusters (**B**) and AChR intensity per unit length of nerve-muscle contacts (**C**) in nerve-muscle co-cultures.

(**D**) Representative images showing the significant inhibitory effects of MβCD pre-treatment on agrin bead-induced AChR clustering upon stimulation for 0.5 and 4 hours. 8-bit pseudo-color images highlight the relative fluorescence intensity of agrin bead-induced AChR clusters under different conditions.

(**E**-**F**) Quantification showing the inhibitory effects of MβCD pre-treatment on the percentage of bead-muscle contacts with AChR clusters (**E**) and the normalized fluorescence intensity of bead-induced AChR clusters (**F**).

Scale bars represent 5 μm. Data are mean ± SEM. The numbers indicated in the bar regions represent the total numbers of nerve-muscle contacts (**B**-**C**) and bead-muscle contacts (**E**-**F**) measured from 3 independent experiments. **, **** represent $p \leq 0.01$, and 0.0001 (Student's t-test).

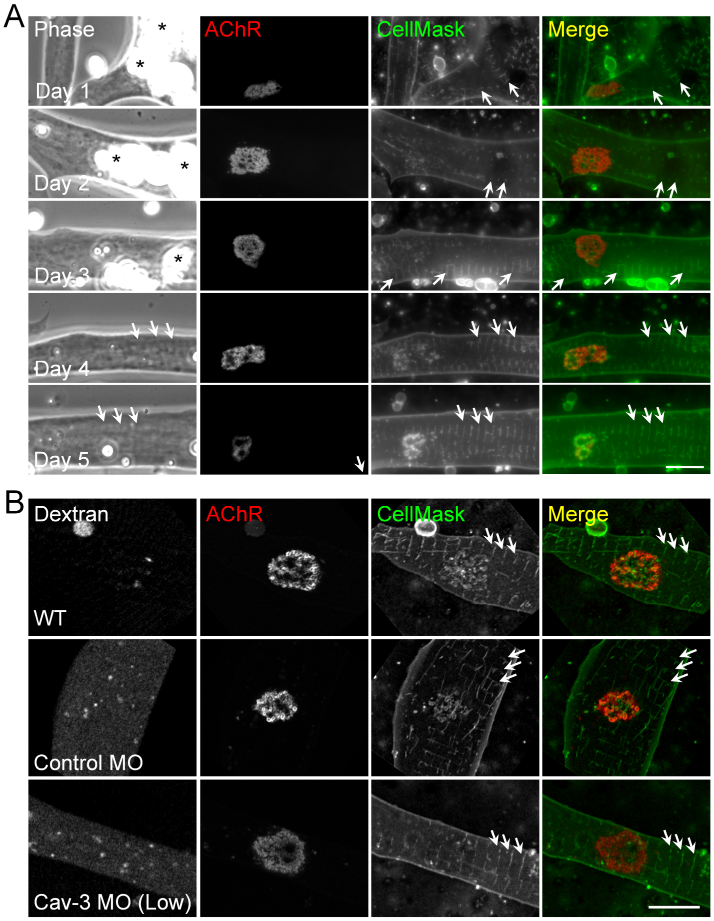

Fig. S9.

Low caveolin-3 knockdown does not affect T-tubule structures in cultured muscle cells.

(**A**) Representative maximal projection of wide-field epifluorescence z-stack images showing the progressive development of T-tubules in cultured muscle cells over the first 5 days in culture. To better show the perforations of aneural AChR clusters, epifluorescence AChR signals were shown from a single focal plane only. Arrows indicate CellMask-labeled sarcolemma invaginations at the T-tubules that can also be observed as striation patterns in phase-contrast images. Asterisks indicate yolk granules inside the cultured muscle cells, which are consumed at the later stage in culture.

(**B**) Representative maximal projection of confocal z-stack images showing no observable difference in T-tubule structures between wild-type, control MO, and low caveolin-3 knockdown muscle cells as shown in Figure 4I. Fluorescent dextran signals indicate the presence of MO. Arrows indicate sarcolemma invaginations at the T-tubules.

Scale bars represent 10 μm.

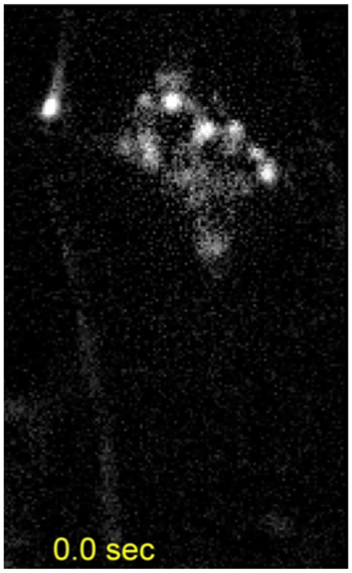

Movie S1.

Time-lapse video of **Fig. 6G** showing that AChR-containing vesicles budded off from the tip of membrane infoldings (red arrowheads) at 38.4 and 47.8 s in agrin bead-stimulated muscle cells. The endocytosed AChR vesicles (red arrows) were subsequently transported toward the site of agrin bead stimulation. Images were taken at 200 ms per frame, and the movie playback speed is 10 frames per second.
